# A marine-derived fungal genome of *Annulohypoxylon annulatoides* reveals AT-rich isochores with putative regulatory functions

**DOI:** 10.1093/g3journal/jkag192

**Published:** 2026-07-13

**Authors:** Che-Yu Thiat-Ut Kuo, Yu-Ching Liu, Cheng-Ju Yang, Chieh-Ping Lin, Hung-Wei Chen, Huei-Mei Hsieh, Yu-Ming Ju, Tsui-Hua Liu, Yi-Chien Lee, Isheng Jason Tsai

**Affiliations:** Biodiversity Research Center, Academia Sinica, Taipei 11529, Taiwan; Taipei Municipal Chien Kuo High School, Taipei 100052, Taiwan; Biodiversity Research Center, Academia Sinica, Taipei 11529, Taiwan; Biodiversity Research Center, Academia Sinica, Taipei 11529, Taiwan; Biodiversity Research Center, Academia Sinica, Taipei 11529, Taiwan; Biodiversity Research Center, Academia Sinica, Taipei 11529, Taiwan; Institute of Plant and Microbial Biology, Academia Sinica, Taipei 11529, Taiwan; Institute of Plant and Microbial Biology, Academia Sinica, Taipei 11529, Taiwan; Taipei Municipal Chien Kuo High School, Taipei 100052, Taiwan; Biodiversity Research Center, Academia Sinica, Taipei 11529, Taiwan; Biodiversity Research Center, Academia Sinica, Taipei 11529, Taiwan

**Keywords:** AT-rich isochores, Hypoxylaceae, genome organization, *Annulohypoxylon*, comparative genomics

## Abstract

Marine and coastal fungi experience intense environmental variability, yet the genomic features associated with tolerance to such conditions remain unclear. From 56 fungal isolates collected along the Lailai rocky shore in northern Taiwan, we selected the coastal isolate *Annulohypoxylon annulatoides* RYS0019 for phenotypic and genomic investigation because of its prevalence and distinctive stress-response profile. Compared with 5 bark-derived conspecific strains, RYS0019 showed distinct growth and recovery dynamics under salinity, temperature, and UV-associated stress treatments. We generated a high-quality 41.8 Mb de novo genome assembly with 11,523 predicted proteins and compared it with 15 other Hypoxylaceae genomes. Across *Annulohypoxylon* genomes, we identified variably sized and dispersed AT-rich isochores that are repeat-enriched and gene-poor. Despite variation in AT content, core gene content and Pfam domain profiles remained broadly conserved. Most AT-rich isochores were embedded within syntenically conserved regions and showed limited positional conservation across species, supporting recurrent, lineage-specific formation or expansion after species divergence. These regions also exhibit several sequence and structural features consistent with scaffold/matrix attachment regions, raising the possibility that they influence higher-order genome organization or context-dependent regulation. Together, our findings identify repeat-rich genome architecture as a dynamic feature of *Annulohypoxylon* genome evolution and provide a framework for testing how such regions may contribute to fungal environmental flexibility.

## Graphical Abstract

**Graphical Abstract:**
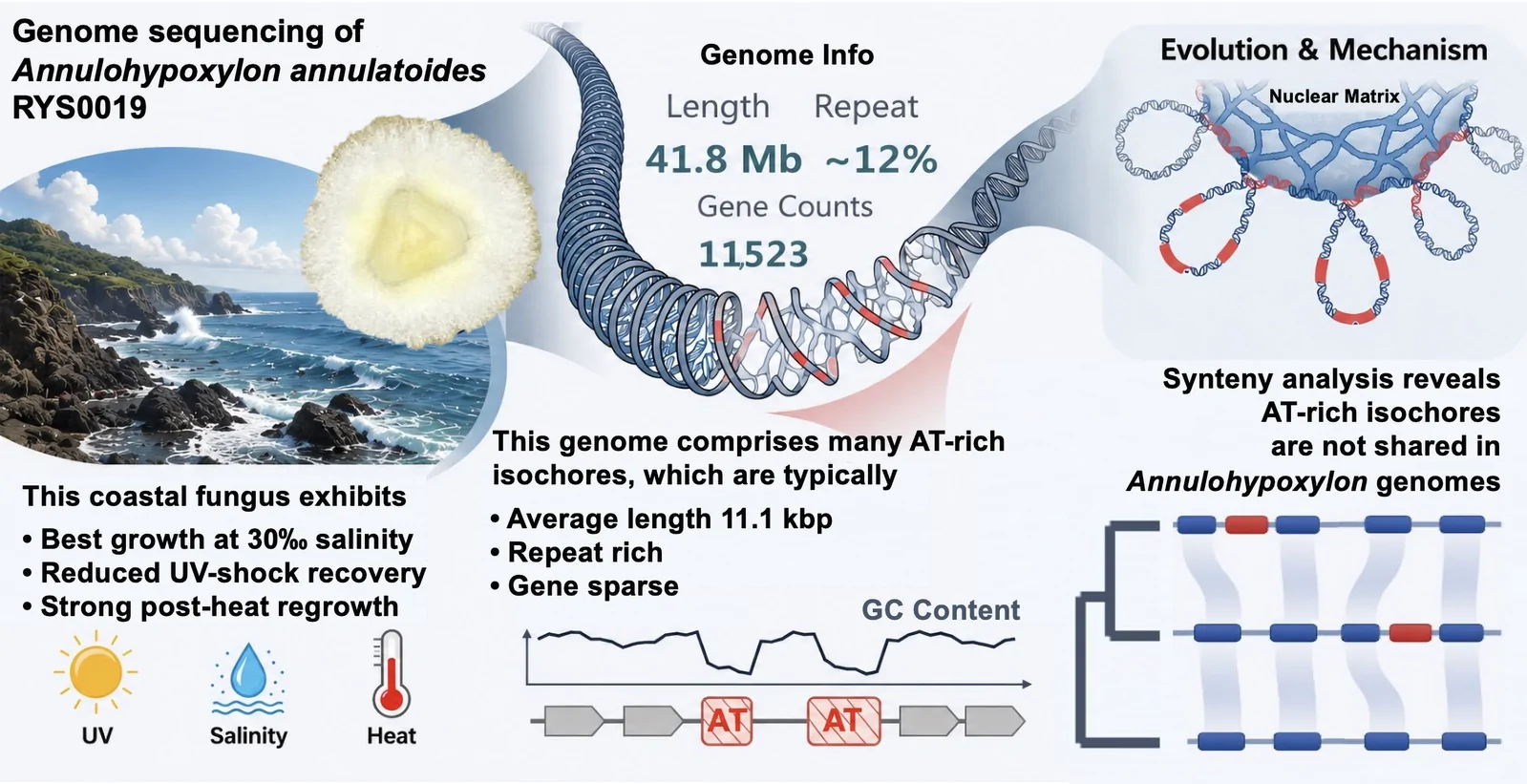

## Introduction

Marine and coastal fungi are increasingly recognized for their ecological roles and biotechnological potential (Bonugli-Santos et al. 2015). Life in the intertidal zone is shaped by sharp swings in solar radiation, salinity, and temperature, demanding tolerance to multiple concurrent stressors (El-Bibany et al. 2014; Pescheck et al. 2014). Among such habitats, rocky shores represent some of the most dynamic and challenging environments, characterized by fluctuating salinity, intermittent desiccation, and intense ultraviolet radiation (Johnson 2024). Ongoing climate change is amplifying thermal, ultraviolet radiation, and osmotic extremes, posing additional challenges to the survival of many species (Bornman et al. 2014; Vargas Zeppetello et al. 2022; Fan and McColl 2024; Huang et al. 2024). Fungal tolerance to such conditions has therefore become a growing focus, particularly with respect to growth under increasingly harsh environments. Rocky shores, in particular, offer a tractable natural system for investigating species tolerance to the extreme environmental conditions expected under future climate change scenarios (Hawkins et al. 2020).

Despite these stressors, many fungi thrive in intertidal habitats. Early research has shown that numerous marine fungi are able to tolerate a wide range of temperatures and high salinity (Ritchie 1957), while others develop thick, rigid, and carbonaceous fruiting bodies that likely buffer against desiccation and UV stress (González and Hanlin 2010). Within this context, members of the family Hypoxylaceae (order Xylariales) stand out. Though typically known as forest endophytes or saprobes in tropical and subtropical regions (U’Ren et al. 2016; Franco et al. 2022), their discovery from Red Sea and mangrove ecosystems reveals their ecological plasticity (Abdel-Wahab 2005). Genomic and transcriptomic studies further indicate that many Hypoxylaceae possess extensive secondary metabolite gene clusters, which may confer their tolerance to stressors (Overy et al. 2019; Franco et al. 2022). In addition, conserved stress-coping genes such as *Sln1*, *Hik1*, and *Sho1* have been identified in multiple Hypoxylaceae genomes (Abdel-Wahab 2005), underscoring a genomic repertoire that may preadapt these fungi to survive the extreme variability of coastal environments.

We hypothesize that fungi inhabiting coastal environments may harbor genomic features and physiological traits associated with repeated exposure to fluctuating stressors, including salinity shifts, thermal extremes, and ultraviolet radiation. To examine this hypothesis, we surveyed the Lailai rocky shore in northern Taiwan and identified *Annulohypoxylon annulatoides* (Hsieh et al. 2024), a member of the Hypoxylaceae. We characterized the phenotypic responses of this coastal isolate in comparison with 5 terrestrial (bark-derived) conspecific strains under varying temperature, salinity, and UV conditions, and generated a high-quality de novo genome assembly. We then compared RYS0019 with other Hypoxylaceae genomes, with a focus on genome evolution across *Annulohypoxylon*. Importantly, while coastal habitats provide a strong ecological motivation for this study, this comparative approach allows us to identify putatively environment-associated patterns from deeper lineage-wide features of genome evolution. Together, our analyses provide a framework for investigating how repeat-driven genome architecture evolves under environmental pressure and may contribute to ecological flexibility across fungal lineages.

## Materials and methods

### Fungal isolation, culture, and strain identification

Fungal samples were collected from Lailai using sterile cotton swabs, with 1 swab used per rock part. Each swab was immediately inoculated onto yeast extract–peptone–dextrose (YPD) agar supplemented with 30‰ salinity. Plates were incubated at 20 °C until visible growth was observed. Individual colonies were then isolated, subcultured onto fresh YPD plates, and routinely maintained at 20 °C in the dark. Isolates that exhibited slow growth at 20 °C were subsequently transferred to 30 °C to facilitate optimal growth.

For strain identification, fresh mycelium from *A. annulatoides* cultures was scraped from the agar surface for genomic DNA (gDNA) and RNA isolation. Fungal DNA was extracted using both QuickExtract and the Quick-DNA Miniprep Kit, following manufacturers’ protocols. The internal transcribed spacer (ITS) region was amplified by PCR using ITS1F (5′-CTTGGTCATTTAGAGGAAGTAA-3′) (Gardes and Bruns 1993) and ITS4 (5′-TCCTCCGCTTATTGATATGC-3′) (White et al. 1990) primers and Taq polymerase. PCR amplification was performed with an initial denaturation at 95 °C for 3 min, followed by 30 cycles of denaturation at 95 °C for 30 s, annealing at 52 °C for 30 s, and extension at 72 °C for 60 s, with a final extension at 72 °C for 5 min. When ITS amplification was unsuccessful, alternative sequences were amplified using primer pairs Bt2a (5′-GGTAACCAAATCGGTGCTGCTTTC-3′) and Bt2b (5′-ACCCTCAGTGTAGTGACCCTTGGC-3′), targeting the beta-tubulin gene (Glass and Donaldson 1995) or CMD5 (5′-CCGAGTACAAGGAGGCCTTC-3′) and CMD6 (5′-CCGATAGAGGTCATAACGTGG-3′), targeting the calmodulin gene (Hong et al. 2005). PCR amplification was performed with an initial denaturation at 95 °C for 5 min, followed by 30 cycles of denaturation at 95 °C for 30 s, annealing at 50 °C (Bt2a/Bt2b) and 55 °C (CMD5/CMD6) for 30 s, and extension at 72 °C for 60 s, with a final extension at 72 °C for 5 min. PCR products were verified by agarose gel electrophoresis, subjected to Sanger sequencing, and the resulting sequences were compared against reference databases to confirm species identity, and genus-level assignments were determined based on the top 10 hits (Supplementary Table 1). ITS sequences were aligned using MAFFT (Katoh and Standley 2013), and phylogenetic relationships were inferred using IQ-TREE (v2.3.5; Minh et al. 2020). The resulting tree was visualized and edited using FastTree (Price et al. 2009).

### Strain phenotyping

Six *A. annulatoides* strains were analyzed, comprising 5 terrestrial isolates (YMJ2247 and YMJ2249-YMJ2252) obtained from tree bark at distinct locations across Taiwan (Hsieh et al. 2024) and 1 coastal isolate, RYS0019, collected from rocky substrate at the Lailai rocky shore. To characterize phenotypic variation between marine and terrestrial strains, we employed 2 complementary approaches to quantify growth performance across a range of environmental conditions.

In the first approach, we examined the effects of temperature (20, 30, and 40 °C) and salinity (20‰, 30‰, and 70‰) by culturing strains on potato dextrose agar (PDA) and manually measuring colony diameters at 2-d intervals. Each strain–condition combination was assayed with 3 biological replicates.

In the second approach, we captured fine-scale growth dynamics using an automated imaging and analysis pipeline. Colonies were scanned every 12 h using an Epson V850 scanner, and hyphal expansion was quantified with PyPhe (v0.98; Kamrad et al. 2020). Images were pre-processed in Photoshop, and hyphal area was segmented and quantified in FIJI using the Trainable Weka Segmentation plugin (Schindelin et al. 2012), trained and validated on 140 images each, achieving 98.33% classification accuracy. From these time-series data, maximum growth rates and carrying capacities were estimated in R.

The same automated workflow was applied to assess responses to acute environmental stress. For heat shock, cultures were grown at 30 °C for 3 d, exposed to 40 °C for 1 d, and then returned to 30 °C for a further 6 d. For UV shock, cultures were grown at 30 °C for 3 d, subjected to UV irradiation at 30 °C for 1 d, and subsequently transferred to PDA supplemented with 30‰ salt and incubated at 30 °C for 6 additional days. UV irradiation was conducted in a Class II Biological Safety Cabinet (11228 BBC 86) using a germicidal UV-C lamp (Sankyo Denki G30T8; peak wavelength 253.7 nm; UV radiant output 13.4 W). Sample was positioned 50 cm from the lamp surface. The UV dose was estimated to be ∼40.3 J/cm^2^ over 24 h. Colony growth throughout both shock treatments was monitored and quantified using the scanner-based pipeline described above. All assays were performed with 3 biological replicates per strain and condition. In the analysis, carrying capacity (*K*) describes the upper asymptote of the growth curve and was estimated from logistic model fitting of area measurements across time. Maximum growth rate (*r*) was defined as the steepest increase in area between consecutive time points.

### Library preparation and sequencing

Fresh mycelium from *A. annulatoides* cultures was collected from agar media for gDNA and RNA extraction. High-molecular-weight gDNA for Oxford Nanopore sequencing was isolated using a modified CTAB-based protocol, involving liquid nitrogen grinding, sequential chloroform extraction in the presence of isoascorbic acid, isopropanol precipitation, and RNase treatment as required, followed by DNA size selection and purification. gDNA for Illumina (for *A. annulatoides* strain RYS0019, YMJ2251, and YMJ2252) sequencing was extracted using the Quick-DNA Microprep Kit.

The genome of *A. annulatoides* RYS0019 was sequenced using a combination of Oxford Nanopore long reads and Illumina short reads (BioProject PRJNA1381326). Oxford Nanopore sequencing was performed using a DNA library prepared with the ligation sequencing kit (SQK-LSK114; Oxford Nanopore Technologies, UK) and sequenced on an R10.4.1 flow cell (FLO-MIN114). Illumina short-read sequencing was outsourced for library preparation using the Illumina DNA PCR-Free Prep kit and sequenced on a NovaSeq X Plus platform. For gene model prediction and genome annotation, total RNA was extracted from *A. annulatoides* cultures using the Qiagen RNeasy Plant Mini Kit. Poly(A)-selected RNA libraries were prepared and sequenced on the Illumina platform.

### Genome assembly

Oxford Nanopore reads were assembled de novo using Flye (v2.9.5; Kolmogorov et al. 2019) assembler. The resulting assemblies were first polished with Medaka (v1.11.3; https://github.com/nanoporetech/medaka) using Nanopore reads to improve consensus accuracy. Redundant haplotigs and overlapping contigs were subsequently identified and removed with Purge_Dups (v1.2.6; Guan et al. 2020). The curated assemblies were further refined by incorporating Illumina short reads using NextPolish (v1.4.1; Hu et al. 2020). Genome completeness was assessed with compleasm (v0.2.6; Huang and Li, 2023) using the ascomycota_obd10 fungi dataset.

### Phylogenetic tree

Protein sequences from *Hypoxylon* sp. FL1284 (Franco et al. 2022), *Hypoxylon fragiforme* CBS 206.31, *Hypoxylon* sp. FL0890, *Hypoxylon* sp. FL0543, *Daldinia vernicosa* CBS 139.73, *Annulohypoxylon minutellum* CBS 135445, *Annulohypoxylon bovei* var. *microspore* CBS 124037, *Annulohypoxylon nitens* CBS 120705, *Annulohypoxylon stygium* TJAS01, *A. stygium* FL0470, *Annulohypoxylon* sp. FPYF3050 (Zhou et al. 2024), *Rostrohypoxylon terebratum* CBS 119137, *Annulohypoxylon truncatum* CBS 140777, *Annulohypoxylon moriforme* CBS 123579, *Annulohypoxylon maeteangense* CBS 123835, and *Xylaria venustula* FL0490 (Supplementary Table 2) were retrieved from JGI Mycocosm (Grigoriev et al. 2014) and NCBI for phylogenetic reconstruction. Orthologous gene sets were identified using OrthoFinder (v2.5.5; Emms and Kelly 2019). Concatenated amino acid alignments of single-copy orthologues were used to infer a maximum-likelihood phylogeny with IQ-TREE (v2.3.5; Minh et al. 2020).

### Repeat masking and gene annotation

In the genome assemblies of *A. stygium* TJAS01, *Annulohypoxylon* sp. FPYF3050, *A. maeteangense*, and *A. annulatoides*, tandem repeats were identified and soft-masked using Tandem Repeats Finder (v4.09.1; Benson 1999). Additional classes of repetitive elements were detected and masked with EarlGrey (v4.2.4; Baril et al. 2024). Gene prediction was performed using BRAKER (v3.0.8; Gabriel et al. 2024), with the *A. annulatoides* hardmasked genome assembly, corresponding protein sequences, and RNA-seq data serving as inputs to train GeneMark-ETP (v1.0; Brůna et al. 2024) and AUGUSTUS (v3.5.0; Stanke et al. 2008). RNA-seq reads were mapped to the genome assembly using STAR (v2.7.11; Dobin et al. 2013), and the resulting alignments were employed to refine GeneMark. Protein files were retrieved from JGI Genome Portal (https://genome.jgi.doe.gov/portal/), including *A. bovei* var. *microspora*, *A. nitens*, *A. stygium*, *A. truncatum*, *A. moriforme*, and *A. maeteangense*, providing additional support for the prediction process.

Functional annotations of the amino acid sequences were carried out using eggNOG-mapper v2 (v2.1.2; default parameters; Huerta-Cepas et al. 2019) on eggNOG v5.0 database. Protein domains and families were identified using pfam_scan (v1.6; http://ftp.ebi.ac.uk/pub/databases/Pfam/Tools/) against the Pfam database (release 37.2; Mistry et al. 2021). Protein sequence of *Hypoxylon* sp. FL1284, *H. fragiforme*, *H.* sp. FL0890, *H.* sp. FL0543, *D. vernicosa*, *A. minutellum*, *A. bovei* var. *microspora*, *A. nitens*, *A. stygium* TJAS01, *A.* sp. FPYF3050, *R. terebratum*, *A. truncatum*, *A. moriforme*, *A. maeteangense*, *X. venustula*, *Xylariaceae* sp. FL0821, *Xylaria digitata* 919, *Entoleuca mammata* CFL468 (JGI CSP Proposal ID: 1999; doi:10.46936/10.25585/60001064), *Biscogniauxia nummularia* (JGI CSP Proposal ID: 2588; doi:10.46936/jejc.proj.2015.49027/60005779), *Fusarium avenaceum* MPI-SDFR-AT-0044 (JGI CSP Proposal ID: 1974; doi:10.46936/10.25585/60001060), *Kretzschmaria zonata* GFP132 (JGI CSP Proposal ID: 1974; doi:10.46936/10.25585/60001060), *Chaetomium tenue* MPI-SDFR-AT-0079 (JGI CSP Proposal ID: 1974; doi:10.46936/10.25585/60001060), and *Nemania serpens* CBS 679.86 (Grigoriev et al. 2014) were used in comparison. We performed a Wilcoxon rank-sum test comparing domain counts between a defined Hypoxylaceae group (including *A. annulatoides*) and outgroup Ascomycota strain. Domains with Benjamini–Hochberg adjusted *P* < 0.05 were considered significantly enriched. The resulting domain abundance matrix was filtered to retain only enriched domains, and species labels were manually curated for downstream visualization. Carbohydrate-active enzymes (CAZymes) were annotated using run_dbcan (v2.0.11; https://github.com/linnabrown/run_dbcan).

### Genome analysis

Genome synteny was analyzed among 4 *Annulohypoxylon* genomes based on orthologous gene relationships. Orthogroups were identified using OrthoFinder (v2.5.5; Emms and Kelly 2019), and single-copy orthologues were extracted for pairwise species comparisons. Protein sequences from each species pair were compared using BLASTP (v2.15.0; Altschul et al. 1990), retaining only reciprocal single-copy orthologues. Syntenic blocks were subsequently inferred using DAGchainer (v1.12; Haas et al. 2004), considering the linear order of orthologous genes along each genome while ignoring physical distance between loci.

AT-rich isochores were identified using OcculterCut segmentation (Testa et al. 2016). Each genome was segmented into compositionally homogeneous regions based on Jensen–Shannon divergence. A species-specific GC-content threshold was then defined from the local minimum separating GC-equilibrated and AT-rich segment distributions. Segments below this threshold and ≥1,000 bp were classified as AT-rich isochores. Downstream analyses focused on *Annulohypoxylon* species harboring more than 1 Mb of AT-rich sequence, as genomes with lower totals are more likely to reflect centromeric regions or insufficient assembly contiguity for robust inference. This criterion included all members of the clade comprising *A. annulatoides*, *A. moriforme*, and *A. maeteangense*, as well as *Annulohypoxylon* sp. FPYF3050 and *A. truncatum* from the sister clade, with *A. bovei* var. *microspora* included as an outgroup.

Genome-wide DNA methylation patterns in *A. annulatoides* were characterized using Oxford Nanopore reads. Raw FASTA files were processed with Dorado (v0.8.3; https://github.com/nanoporetech/dorado) and Modkit (v0.4.1; https://github.com/nanoporetech/modkit) under default parameters to detect 5-methylcytosine (5mC) and *N*^6^-methyldeoxyadenosine (6mA). Methylation calls supported by fewer than 10 reads or with posterior probabilities below 10% were excluded. Methylation levels were summarized in non-overlapping 10 kb windows across the genome.

Promoter-associated motif density was assessed using FIMO (Grant et al. 2011) with fungal transcription-factor-binding profiles from the JASPAR database (Ovek Baydar et al. 2026). Motif density was calculated as the number of motif matches per kilobase. AT-rich isochores were compared with 1-kb random genomic regions sampled from the non-AT-rich genomic background. Because these motifs were computationally predicted, they were interpreted as candidate promoter-associated regulatory motifs rather than experimentally validated promoters.

To compare AT-rich isochore representation among *A. annulatoides* strains, Illumina reads from RYS0019, YMJ2251, and YMJ2252 were mapped to the RYS0019 reference genome using bwa-mem2 (Li 2013; Vasimuddin et al. 2019). Mean read coverage was calculated by Mosdepth (Pedersen and Quinlan 2018) for each AT-rich isochore and normalized by the sample-specific length-weighted mean coverage across all AT-rich isochores. Regions with normalized coverage greater than 100-fold were excluded from visualization to avoid distortion by extreme outliers.

## Results

### Hypoxylaceae dominates the culturable fungal assemblage at the Lailai rocky shore environment

**Fig. 1.**
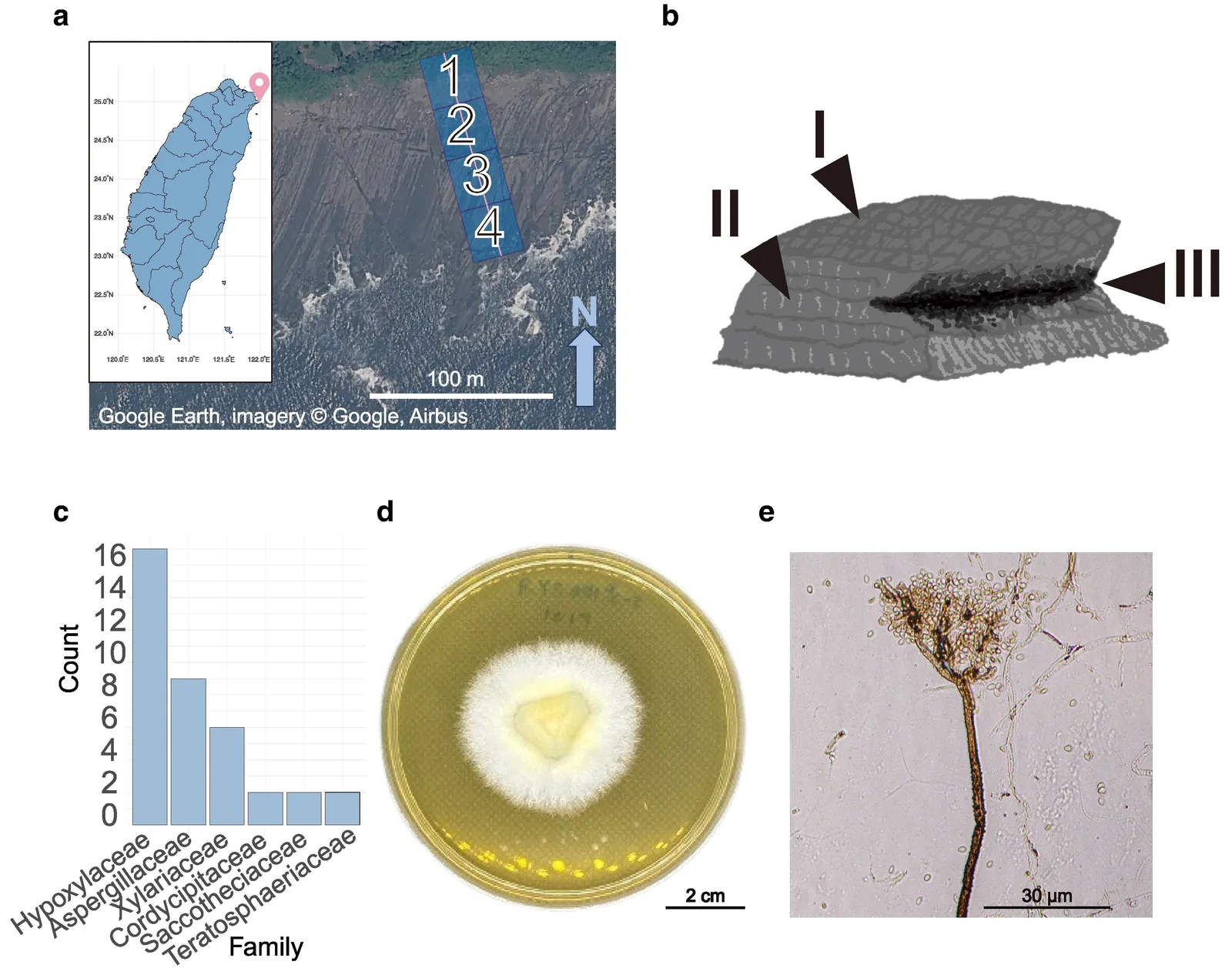
Sampling design, fungal diversity, and morphology of *A. annulatoides* RYS0019. a) Aerial view of the Lailai rocky shore showing 4 sampling areas along a transect from the vegetation line (area 1) to the coastline (area 4) (photo: Cian-siang You, 2015). b) Schematic of the 3 rock microhabitats sampled: (i) upper surface, (ii) lateral side, and (iii) crevice. c) Number of isolates per fungal family, including only families represented by more than 2 isolates. d) Colony morphology and mycelial growth, and (e) conidiophore formation of *A. annulatoides* RYS0019 cultured on PDA at 30 °C.

We established 4 contiguous 25 × 25 m plots along the wave-cut platform of the Lailai rocky shore in Northern Taiwan (areas 1 to 4; Fig. 1a), spanning a gradient from the terrestrial vegetation line to the seaward edge. Within each area, 3 rock surfaces were randomly selected, and from each rock we sampled 3 distinct microhabitats—the upper surface, lateral sides, and rock crevices (Fig. 1b). A total of 56 fungal isolates were obtained and identified to at least the genus level based on sequencing of the ITS region (Supplementary Table 1). Hypoxylaceae accounted for the majority of isolates (Fig. 1c), suggesting this family dominates the culturable fungal assemblage at the Lailai rocky shore. Amongst the recovered taxa, *Annulohypoxylon* (3 isolates), *Hypoxylon* (10 isolates), *Penicillium* (9 isolates), and *Whalleya* (5 isolates) were most frequently recovered.

Spatial comparisons showed areas 1 and 4 yielded the highest number of isolates (16 each) yet exhibited distinct taxonomic profiles (Supplementary Fig. 1a), likely reflecting environmental gradients along the transect. Area 1 bordered terrestrial vegetation, whereas area 4 was directly exposed to the shoreline. We obtained the greatest number of isolates from rock crevices (22 isolates, Supplementary Fig. 1b). Hypoxylaceae species were consistently recovered across all areas and microhabitats, making Hypoxylaceae the dominant culturable fungal group in this intertidal assemblage. Building on these observations, we selected a representative coastal *A. annulatoides* isolate, RYS0019, for phenotypic and genomic analysis (Fig. 1d and Supplementary Fig. 2).

### Divergent stress recovery patterns in the coastal strain RYS0019 of *A. annulatoides*

**Fig. 2.**
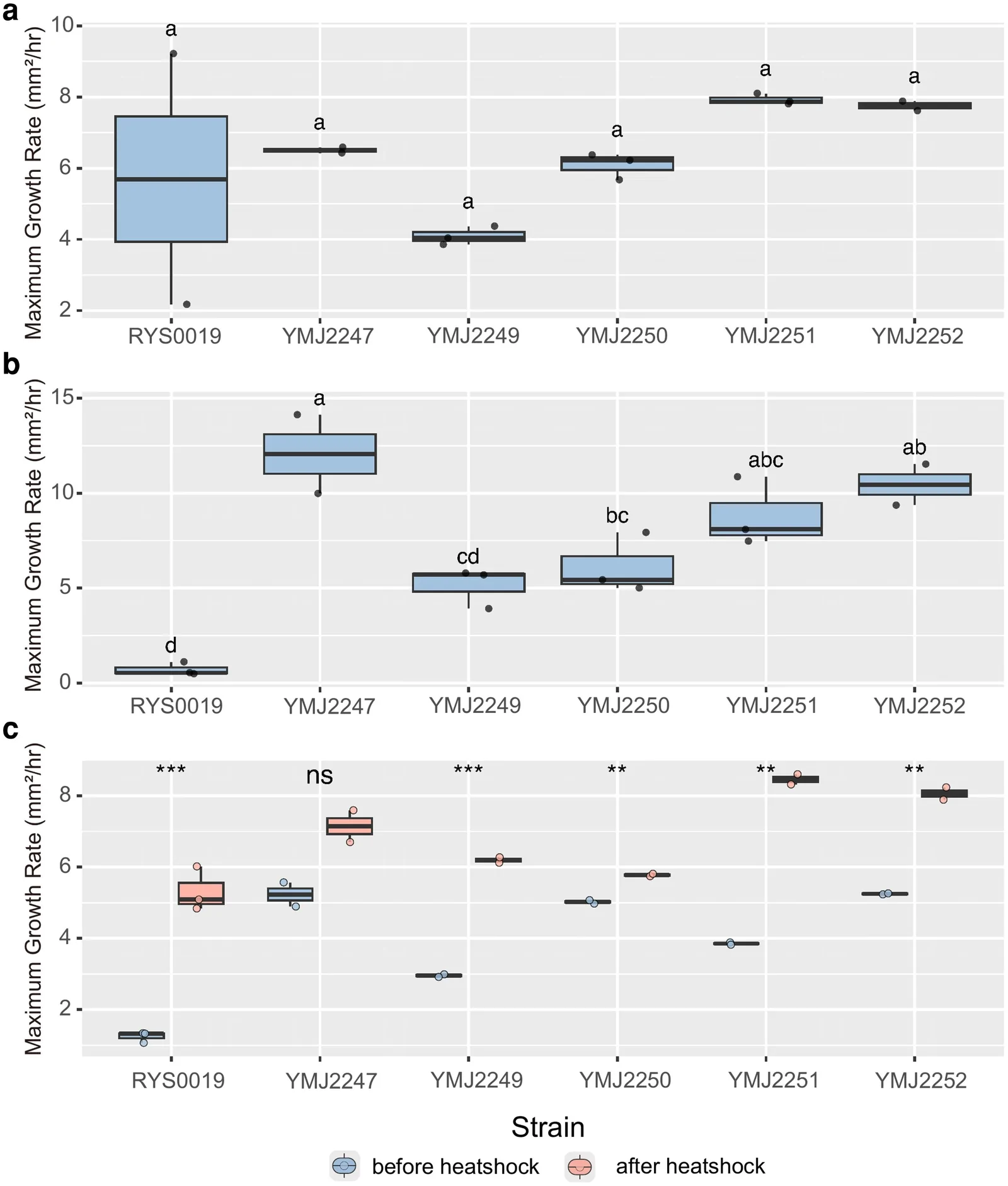
Growth performance of *A. annulatoides* strains under baseline and stress conditions. Maximum growth rates of 6 *A. annulatoides* strains measured under (a) standard growth at 30 °C in the dark, (b) after 1 d of UV shock followed by growth at 30 °C in the dark, and (c) before and after heat shock, in which cultures were exposed to 40 °C and then returned to 30 °C in the dark. Boxplots show median, interquartile range, and individual biological replicates. Different letters indicate significant differences among strains within each condition based on 1-way ANOVA followed by Tukey's post hoc test; asterisks denote significant differences between pre- and post-heat-shock treatments (ns, not significant; **P < 0.01; ***P < 0.001).

We evaluated the stress tolerance of 6 *A. annulatoides* strains isolated from contrasting environments under varying temperature, salinity, and UV exposure conditions. Growth at 20 and 40 °C was measured based on colony diameter, whereas other phenotypic assays were evaluated using a scanner by quantifying the mycelial area on plates. All raw growth data are provided in Supplementary Tables 3 and 4. At a constant 30 °C, bark-derived strains showed similar growth profiles (on average *r* = 5.40 mm^2^/h and *K* = 549.00 mm^2^), whereas the coastal strain RYS0019 exhibited a comparable maximum growth rate (*r* = 5.69 mm^2^/h) but a lower carrying capacity (*K* = 408.96 mm^2^) (Fig. 2a). None of the strains grew at 40 °C (Supplementary Figs. 3 and 4). Following UV exposure, all terrestrial strains recovered to near-control levels, whereas RYS0019 showed a pronounced decline in carrying capacity (*K* = 52.79 mm^2^) and maximum growth rate (*r* = 0.71 mm^2^/h) (Fig. 2b and Supplementary Fig. 5). Under saline conditions, RYS0019 achieved its highest carrying capacity at 30‰, in contrast to other strains that showed reduced or unchanged growth at this concentration compared with growth under 0‰ salinity (Supplementary Figs. 4 and 6). These results indicate that strain RYS0019 exhibits a distinct stress-response profile compared with the bark-derived strains, showing its highest carrying capacity under moderate salinity but slower growth and recovery following UV-associated stress. This pattern may reflect a more conservative strategy of resource allocation, in which growth is reduced in order to better tolerate or persist under stressful conditions over the longer term.

Building on its divergent stress responses, and observing that the ambient temperature at the time of sampling reached 32.3 °C, we next assessed whether RYS0019 exhibited a characteristic pattern of thermal recovery. Strains pre-grown under standard conditions were subjected to a 1-d 40 °C heat shock. All strains showed reduced growth during heat exposure, followed by increased growth rates post-treatment (Fig. 2c). In particular, RYS0019 displayed the most pronounced recovery, with its maximum growth rate increasing from 1.24 to 5.31 mm^2^/h, potentially reflecting a strategy of rapid regrowth after transient stress, although differences in growth phase may also contribute to the observed change. As all *A. annulatoides* strains are generally resilient to environmental fluctuation, these patterns may indicate that RYS0019 prioritizes post-stress recovery over maximal resistance, consistent with an environment characterized by frequent but transient stress episodes rather than sustained extremes.

### Genome assembly and annotation of *A. annulatoides* RYS0019

To investigate genomic features associated with the coastal isolate RYS0019, we performed whole-genome sequencing using Oxford Nanopore long-read technology. The initial assembly was produced with Flye (Kolmogorov et al. 2019), polished with Oxford Nanopore reads using Medaka, and subsequently polished with Illumina reads using NextPolish (Hu et al. 2020), resulting in a 41.8 Mb genome assembly comprising 28 contigs with N50 of 4.4 Mb (Supplementary Table 2). Three contigs contained TTAGGG copies at both ends and 7 contained TTAGGG copies at 1 end (Supplementary Table 5). Gene prediction with the BRAKER3 (Gabriel et al. 2024) pipeline aided by reference protein homology support of closely related species and transcriptome sequencing identified 11,523 protein-coding gene models. Assembly completeness was estimated at 97.5% using Compleasm (Huang and Li 2023), exceeding 3 other sequenced *Annulohypoxylon* species (Supplementary Table 2). Orthologue clustering with OrthoFinder (Emms and Kelly 2019) on 16 Hypoxylaceae proteomes and *X. venustula* FL0490 as outgroup identified 12,686 orthologous groups, including 1,145 single-copy orthologues. 1,607 orthogroups were shared across all Hypoxylaceae species, while 119 were unique to Hypoxylaceae compared with outgroup *X. venustula* FL0490.

**Fig. 3.**
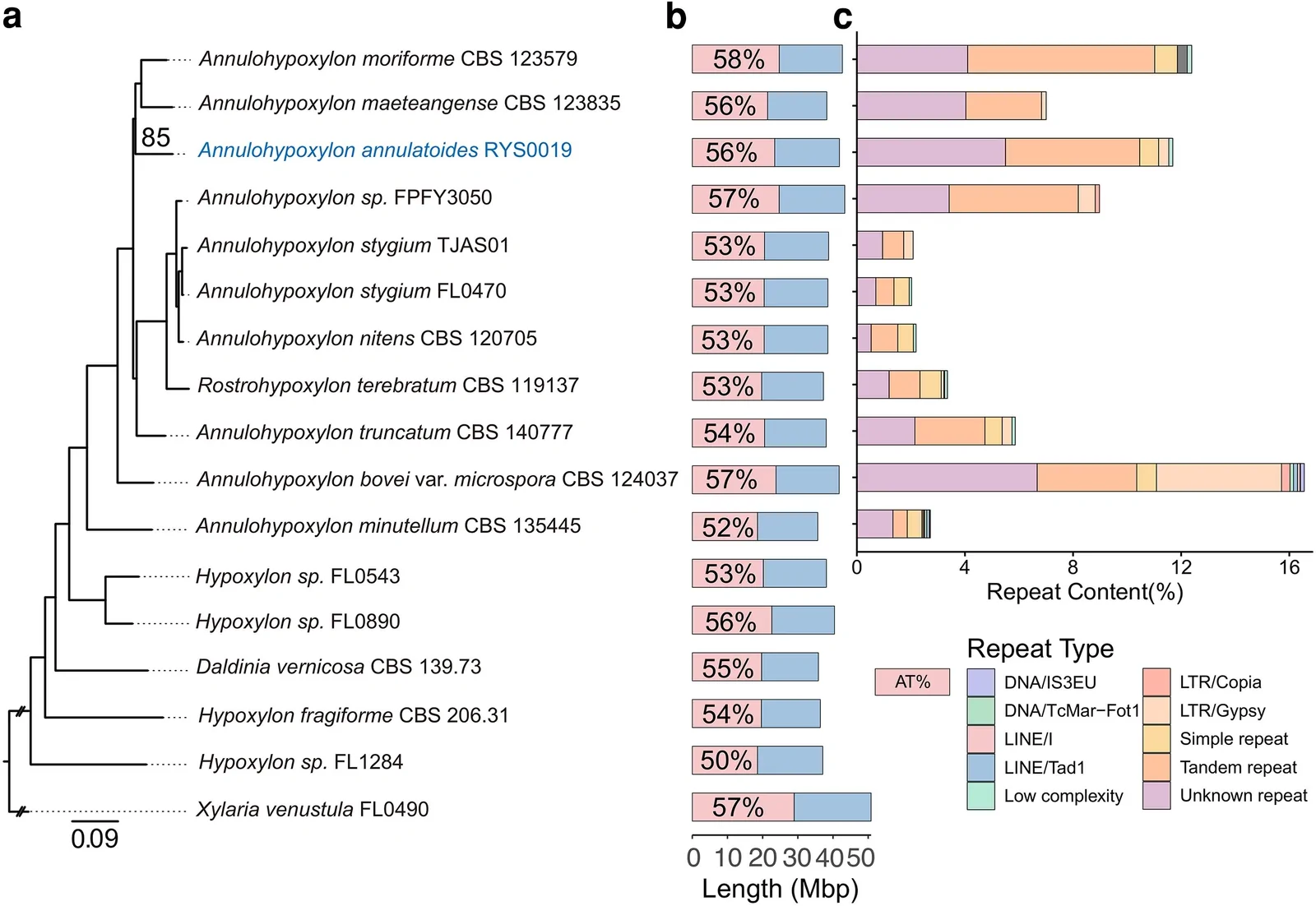
Phylogeny, genome size, and AT-rich repeat composition in Hypoxylaceae. a) Phylogenetic relationships among 16 Hypoxylaceae species, rooted with *X. venustula* FL0490 as the outgroup. All branches except the annotated branch have bootstrap support values of 100. b) Genome size and overall AT content for each species. c) Composition and relative abundance of major repeat classes across 11 *Annulohypoxylon* genomes.

To determine the phylogenetic placement of *A. annulatoides*, we constructed a maximum-likelihood phylogeny based on a concatenated alignment of 1,144 single-copy orthologues (Fig. 3a) and a coalescent-based phylogeny of individual gene trees (Supplementary Fig. 7). In the concatenated alignment of 1,144 single-copy orthologues, *A. annulatoides* RYS0019 formed a well-supported clade with *A. maeteangense* CBS 123835 and *A. moriforme* CBS 123579. This clade was resolved as sister to another robust *Annulohypoxylon* group comprising *A. stygium* TJAS01, *A. nitens* CBS 120705, *A.* sp. FPYF3050, and *R. terebratum* CBS 119137, together delineating 2 major lineages within the broader *Annulohypoxylon*-related group. The coalescent-based phylogeny inferred from individual gene trees recovered a largely similar topology, with the main difference involving *A. truncatum* CBS 140777, which was placed as sister to the *A. annulatoides–A. maeteangense–A. moriforme* clade. Overall, the concatenated phylogeny was consistent with previous phylogenomic analyses of Xylariales (Franco et al. 2022), supporting the stability of these deep relationships within Hypoxylaceae.

Within the *Annulohypoxylon* clade, *A. annulatoides* possesses one of the largest genomes (41.8 Mb; Fig. 3b), and AT content of 56%. *A. moriforme* and *A. maeteangense* formed a sister group with *A. annulatoides*, all of which possessed relatively large genomes ranging from 38.2 to 42.7 Mb and high AT contents (56% to 58%) (Fig. 3b). The sister clade, comprising *A. stygium* TJAS01, *A. stygium* FL0470, and *A. nitens*, exhibited genome sizes between 38.5 and 38.8 Mb, with lower AT contents of 53%. *A. truncatum* also belonged to this group, showing a genome size of 38.1 Mb and an AT content of 54%. Outside these 2 lineages, *A. bovei* var. *microspora* displayed a genome size of 41.8 Mb and an AT content of 57%, whereas *A. minutellum*, the most basal species in the phylogeny, had the smallest genome (35.7 Mb) and the lowest AT content (52%).

### Repeat content and genome size

To account for the relatively larger genome size observed in *A. annulatoides*, we conducted repeat annotation across *Annulohypoxylon* genomes (Fig. 3c). Known repeats such as transposable elements (TEs) were not the predominant components. Instead, unknown and tandem repeats constituted the majority. In *A. annulatoides*, repetitive sequences spanned ∼12% of the genome, with tandem and unknown repeats comprising ∼11%, representing the highest proportions among all examined species. Similarly, several species with larger genomes including *A. maeteangense*, and *A.* sp. FPYF3050 also harbored elevated proportions of these repeat classes (6% to 11%).

Repeat content was positively correlated with genome size (Pearson's *r* = 0.78, *P* < 0.001; Supplementary Fig. 8), indicating that repeat accumulation contributes substantially to genome expansion in this genus. AT content analyses revealed that repeats were consistently AT-rich across all species (Supplementary Fig. 9). Accordingly, genomes with greater repeat loads—including *A. annulatoides*, *A. maeteangense*, and *A.* sp. FPYF3050—displayed higher overall AT content (56% to 57%), whereas species with fewer repeats, such as *A. stygium* TJAS01, exhibited lower AT content (53%) (Fig. 3c). These patterns collectively indicate that AT-rich repetitive sequences are a major driver of genome expansion in *A. annulatoides* and related species.

### Repeats are mostly AT-rich isochores

Given the strong correlation between repeat abundance and genomic AT content, we next examined whether these repeats tend to accumulate within specific AT-rich compartments. Using OcculterCut segmentation (Testa et al. 2016), we identified extensive AT-rich tracts across all 8 *Annulohypoxylon* genomes, ranging in size from 1,000 to 155,667 bp, with an average length of 11,102 bp—consistent with the hallmarks of AT-rich isochores (Supplementary Fig. 10). The majority of annotated repeats were localized within these regions: 69.9% to 86.8% of all repeats were located within AT-rich isochores, including 21.2% to 52.0% tandem repeats and 21.9% to 47.5% unknown repeats (Supplementary Fig. 11a). In *A. annulatoides*, some of these isochores contained homopolymeric tracts exceeding 29 identical bases (Supplementary Fig. 12). In contrast to their dense repeat load, AT-rich isochores exhibited markedly lower gene coverage—∼14-fold lower than GC-rich regions (Supplementary Fig. 11b), indicating that AT-rich isochores are predominantly intergenic, gene-poor genome compartments.

### Genome conservation and independent isochore origins

**Fig. 4.**
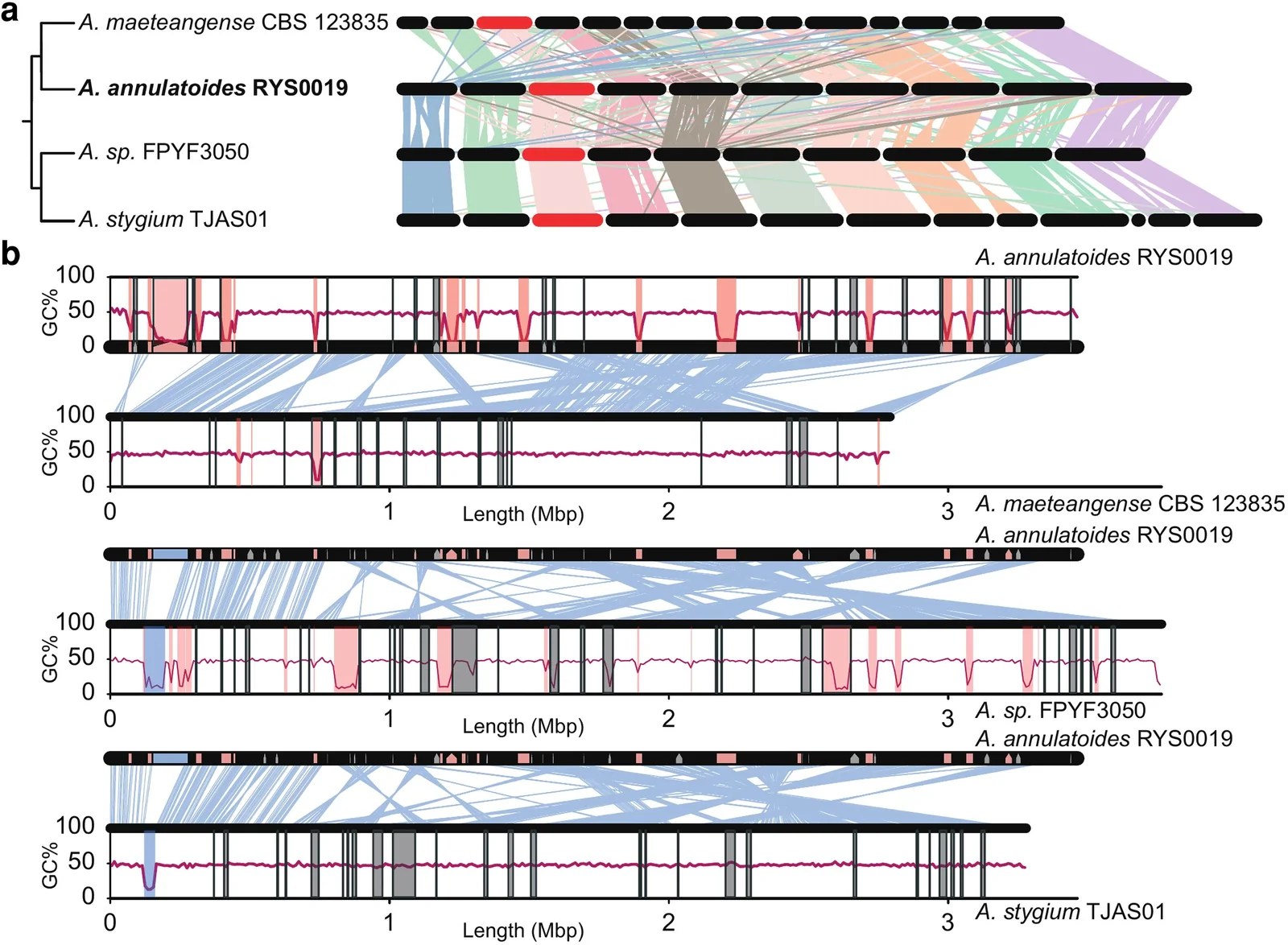
Synteny and intergenic isochore distribution among *Annulohypoxylon* species. a) Syntenic relationships among contigs larger than 1 Mb in *A. maeteangense*, *A. annulatoides*, *A.* sp. FPYF3050, and *A. stygium* TJAS01, defined using single-copy orthologs and shown as blue connecting lines. Red-colored contigs were extracted for (b). b) Distribution of intergenic regions containing isochores in *A. annulatoides* relative to the 3 comparison species. GC content is shown as a red line. Outlined rectangles or pentagons mark intergenic regions at syntenic breakpoints, with grey indicating absence and red indicating presence of isochores. In regions without block outlines, red denotes isochores unique to 1 species, whereas blue indicates isochores shared between corresponding intergenic regions. Genomic coordinates are shown in megabases (Mb).

To assess whether isochores coincide with larger-scale structural changes, we investigated genome synteny among 4 *Annulohypoxylon* species with high-quality assemblies (N50 > 1 Mb). Syntenic blocks were defined as regions in which the order and orientation of orthologous genes between 2 species were preserved, whereas syntenic breaks correspond to intergenic regions separating conserved blocks of orthologous genes. Comparisons between *A. annulatoides* and *A. maeteangense*, *A.* sp. *FPYF3050*, and *A. stygium* TJAS01 revealed that 90.23% to 91.54% of their genomes fell within conserved syntenic blocks, while having 165 to 217 syntenic breaks. Most syntenic blocks were assigned to the same orthologous contigs, indicating that despite the presence of numerous rearrangements, the majority of changes represent intra-chromosomal events (Fig. 4a).

Only a small fraction of breakpoints overlapped isochores: 13.33% in *A. maeteangense*, 12.15% in *A.* sp. *FPYF3050*, and 10.14% in *A. stygium*. Among inversion-associated breakpoints, overlap remained limited: 15.15% in *A. maeteangense*, 10.00% in *A.* sp. *FPYF3050*, and 6.52% in *A. stygium*. In total, just 1.64% to 2.60% of isochores were located at synteny breakpoints, indicating that the vast majority are embedded within regions of conserved gene order rather than associated with structural rearrangements. This pattern is visually evident in Fig. 4, a and b, where isochores frequently appear in intergenic regions flanked by conserved syntenic blocks. In addition, breakpoints containing isochores were substantially longer—averaging 43.63 to 65.65 kb—than those lacking isochores (mean length 9.36 to 11.86 kb), indicating that the presence of AT-rich isochores is associated with local intergenic expansion. These findings suggest that while most isochores arise independently within syntenically stable regions, a subset may insert into structurally dynamic loci and contribute to regional expansion.

**Fig. 5.**
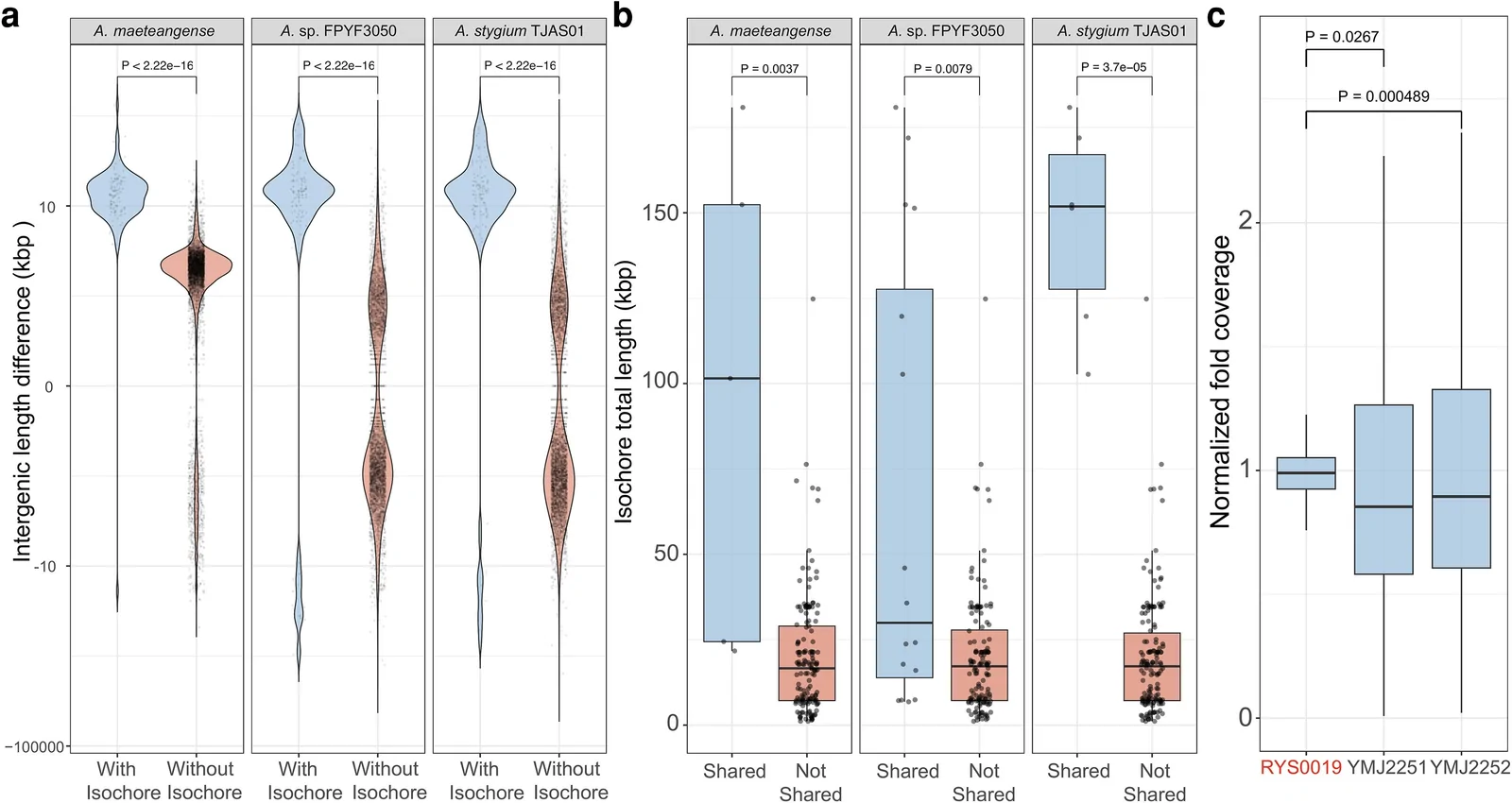
Intergenic expansion associated with AT-rich isochores in *Annulohypoxylon*. a) Comparison of relative intergenic lengths of *A. annulatoides* and 3 related *Annulohypoxylon* species, contrasting intergenic regions containing AT-rich isochores with those lacking isochores. b) Total lengths of AT-rich isochores in intergenic regions that are conserved between *A. annulatoides* and each comparison species (shared) vs isochores located in intergenic regions where the corresponding species lacks an isochore (not shared). c) Normalized Illumina read coverage across AT-rich isochore regions in *A. annulatoides* RYS0019 and 2 bark-derived strains, YMJ2251 and YMJ2252. Coverage was normalized by the sample-specific length-weighted mean coverage across all AT-rich isochores; regions with normalized coverage >100-fold were excluded from visualization. *P*-values denote Wilcoxon rank sum tests.

Across species, only 3.16% to 10.27% of isochores were found in the same intergenic regions across species (Supplementary Fig. 13), indicating isochores are highly dynamic within otherwise conserved genome architecture. This is further illustrated in Fig. 4b, where conserved syntenic blocks are preserved across species, but the presence or absence of isochores at corresponding loci varies markedly. In *A. annulatoides*, *A. maeteangense*, *A.* sp. *FPYF3050*, and *A. stygium*, intergenic regions consisting of isochores were substantially longer than other orthologous intergenic regions (mean length 27.07 to 29.12 vs 1.95 to 2.08 kb; Fig. 5a). Consistent with this, 76.09%, 87.50%, and 87.83% of intergenic regions that expanded by more than 10 kb (relative to *A. maeteangense*, *A.* sp. FPYF3050, and *A. stygium* TJAS01) contained AT-rich isochores. In these cases, AT-rich isochores account for 82.23% to 85.71% of the total expansion, confirming that isochores are the primary drivers of large-scale intergenic extension.

A small subset of positionally conserved isochores was markedly longer than the remainder (Fig. 4b). For instance, in comparisons with *A. stygium*, shared isochores had a mean length of 14.64 kb, whereas non-shared isochores averaged only 1.97 kb (Fig. 5b). These unusually long, conserved intergenic intervals are consistent with centromeric or pericentromeric regions that are maintained across species, including lineages that otherwise lack extensive AT-rich isochores. Supporting this interpretation, resequencing analyses revealed pronounced differences in read coverage across isochore regions among *A. annulatoides* strains (Fig. 5c). The terrestrial strains YMJ2251 and YMJ2252 exhibited significantly reduced and more variable normalized coverage over AT-rich isochores relative to the coastal isolate RYS0019 (Wilcoxon rank-sum test, *P* < 0.05), indicating strain-specific divergence in isochore structure or copy number. Together, these results suggest that while a subset of long isochores is positionally constrained, the majority of AT-rich intergenic regions remain evolutionarily dynamic and differ substantially between lineages.

### Spatial and regulatory features of AT-rich isochores

To further investigate the distribution of AT-rich isochores, we examined their positions along contigs. Here, we compared the distance of each isochore from the contig midpoint in *A. annulatoides* and *A.* sp. FPYF3050, the 2 species that exhibit strong AT-rich signals and share clear one-to-one contig correspondence. Kernel density estimates were generated to visualize the distribution of AT-rich isochores along contig. In both species, isochores are enriched toward contig ends and are strongly depleted near the midpoint (Supplementary Fig. 14, a and b).

To test whether isochore length varied by chromosomal position, we categorized isochores as near-terminal (distance to contig end ≤0.25) or central (>0.25) and compared their lengths (Supplementary Fig. 14, c and d). In *A. annulatoides*, median lengths between near-terminal and central isochores were nearly identical (15,989 vs 13,871 bp). In *A.* sp. *FPYF3050*, the median was higher in central regions (21,290 bp) compared with terminal regions (11,752 bp), although the difference was not statistically significant (Wilcoxon test, *P* = 0.07). This result indicates that although AT-rich isochores consistently accumulate near chromosome ends across species, their lengths do not differ significantly across chromosomal regions. Despite variation in repeat composition and differences in length, the shared distribution patterns hint at conserved underlying processes.

To explore the functional potential of AT-rich isochores, we examined their spatial organization and regulatory characteristics. These regions also showed a higher density of predicted promoter-associated motifs than the genomic background across all 3 tested species (Supplementary Fig. 15) and exhibited lower levels of DNA methylation than neighboring GC-rich segments in *A. annulatoides* (Supplementary Fig. 16), indicating a more open chromatin configuration. We next examined the basal expression of genes flanking AT-rich isochores using RNA-seq data generated under standard growth conditions. These flanking genes did not show a clear pattern of elevated expression relative to genome-wide expression levels (Supplementary Fig. 17). Although downstream genes showed a statistically significant reduction in expression relative to the genome-wide expression level (0.76-fold change; *P* = 0.001), the magnitude of this difference is small. This suggest that any regulatory influence of these regions is unlikely to reflect broad constitutive activation under standard growth conditions. Instead, their low methylation, motif enrichment, and interspecies variability raise the possibility of context-dependent regulatory roles under specific environmental or developmental conditions.

### Proteome specialization of *A. annulatoides*

We compared the number of genes, protein families, transporters, and CAZymes across Hypoxylaceae species (Supplementary Fig. 18). Protein families that were broadly enriched within Hypoxylaceae showed no further expansion in *A. annulatoides* RYS0019, suggesting that its adaptation does not involve further expansion of these gene families (Supplementary Fig. 19). Several stress-related protein families, however, were consistently enriched in Hypoxylaceae species compared with outgroup species. These included domains such as Ald_Xan_dh_C, associated with oxidative stress tolerance (Malik et al. 2019). Previously characterized stress-responsive genes, such as *Sln1*, *Hik1*, and *Sho1*, were also present in *A. annulatoides* but were not specifically expanded. These genes likely contribute to baseline resilience across both terrestrial and marine environments, though their expression dynamics under environmental stress remain to be investigated. Collectively, these findings suggest that *A. annulatoides* has retained the core genomic repertoire typical of Hypoxylaceae but may involve regulatory or structural genome features rather than major gene-content expansion.

## Discussion

### Phenotypic and genomic features of the coastal isolate RYS0019

Our culture-based survey revealed that Hypoxylaceae species dominated the culturable fungal assemblage at the Lailai rocky shore, consistent with their broad ecological tolerance and previously reported presence in marine environments (Schatz 1988; Leman-Loubière et al. 2017). This culture-based result reflects the composition of the culturable fungal assemblage and provides a starting point for future culture-independent surveys of the broader community. Rock crevices harbored the highest number of Hypoxylaceae isolates (5 isolates; Supplementary Fig. 1b), likely due to their relatively stable microclimatic conditions, including reduced desiccation and lower solar exposure. This occurrence suggests some members of Hypoxylaceae can persist under fluctuating coastal conditions, likely supported by a conserved repertoire of stress-related genes.

The coastal isolate *A. annulatoides* RYS0019 showed a stress-response profile that differed from the 5 bark-derived conspecific strains tested. It exhibited slower baseline growth but showed pronounced recovery after transient heat stress and relatively strong performance under moderate salinity. This pattern may reflect strain-level physiological variation in growth and recovery under fluctuating conditions—a strategy commonly observed among fungi in extreme environments (Gostinčar et al. 2022). Additional coastal and terrestrial *A. annulatoides* isolates will be needed to determine whether the stress-response profile observed in RYS0019 is consistently associated with rocky-shore environments or reflects strain-level variation.

### Evolutionary turnover of AT-rich isochores across species

RYS0019 contains extensive AT-rich, repeat-dense regions, but comparative analyses showed that these regions are not unique to this isolate and occur across multiple *Annulohypoxylon* genomes. Despite broadly conserved genome architecture across the genus, AT-rich isochores are highly dynamic: most were embedded within syntenically conserved regions, whereas only a small fraction (1.64% to 2.60%) overlapped synteny breakpoints. These results indicate that repeat-rich genome compartments are a recurrent feature of *Annulohypoxylon* genome evolution and that their formation is largely decoupled from large-scale structural rearrangements. The few isochores that do coincide with structural breakpoints may have inserted into structurally dynamic loci or expanded after rearrangement events, contributing to local intergenic expansion.

Isochores also show minimal positional conservation across species, with only 3.16% to 10.27% occupying homologous intergenic regions. If most AT-rich isochores had been inherited from the most recent common ancestor and subsequently lost in different lineages, we would expect more shared genomic positions and detectable sequence conservation within corresponding AT-rich regions. Instead, most isochores are lineage-specific, repeat-rich, and embedded within otherwise conserved syntenic regions, supporting recurrent, independent formation or expansion after species divergence. Nevertheless, a small subset of isochores is conserved in both position and structure across species. These shared isochores are substantially longer than lineage-specific isochores and may represent ancient elements retained because of functional or structural constraints, potentially corresponding to centromeric, pericentromeric, or otherwise structurally important regions. Thus, *Annulohypoxylon* genomes appear to contain 2 classes of AT-rich isochores: a small set of conserved, structurally constrained regions and a larger set of rapidly evolving, lineage-specific regions generated by repeat-associated expansion.

Taken together, these patterns support a model in which AT-rich isochores evolve through recurrent repeat accumulation within otherwise conserved genome regions. Their limited association with synteny breakpoints argues against the idea that they are simply byproducts of large-scale structural rearrangement. Instead, repeat-driven intergenic expansion appears to be a major mechanism shaping isochore emergence and diversification across *Annulohypoxylon* genomes.

### Possible mechanisms driving AT-rich isochore formation

Across *Annulohypoxylon* genomes, 70% to 87% of annotated repeats were located within AT-rich isochores, indicating that repeat accumulation is the major contributor to their formation. Repeat-rich regions in fungi are often associated with TE activity (Zattera and Bruschi 2022) or structural rearrangements (Xie et al. 2024). However, our synteny analyses show that most AT-rich isochores are embedded within conserved genomic regions and are largely uncoupled from major contig rearrangements. In addition, repeat annotation revealed that these regions are dominated by tandem and unknown repeats rather than canonical TEs, suggesting that their formation may involve mutational processes distinct from TE-driven expansion.

Given their repeat composition, the initial formation of AT-rich isochores is likely seeded by slipped-strand mispairing, a mechanism known to promote tandem repeat expansion (Levinson and Gutman 1987). However, the large size and widespread distribution of these isochores suggest that slippage alone is insufficient to explain their proliferation. Additional processes, such as unequal crossing-over (Smith 1976) or replication-based amplification (Polleys et al. 2017), may further extend and propagate these repeat-rich regions. Acting recurrently across lineages, these mechanisms could generate the dynamic and largely lineage-specific landscape of AT-rich isochores observed across *Annulohypoxylon* genomes.

### Putative function of AT-rich isochores

The widespread presence and lineage-specific turnover of AT-rich isochores in *Annulohypoxylon* genomes suggest that genome architecture may contribute to regulatory or structural variation. We propose 2 non-mutually exclusive mechanisms by which isochores may influence genome function.

**Fig. 6.**
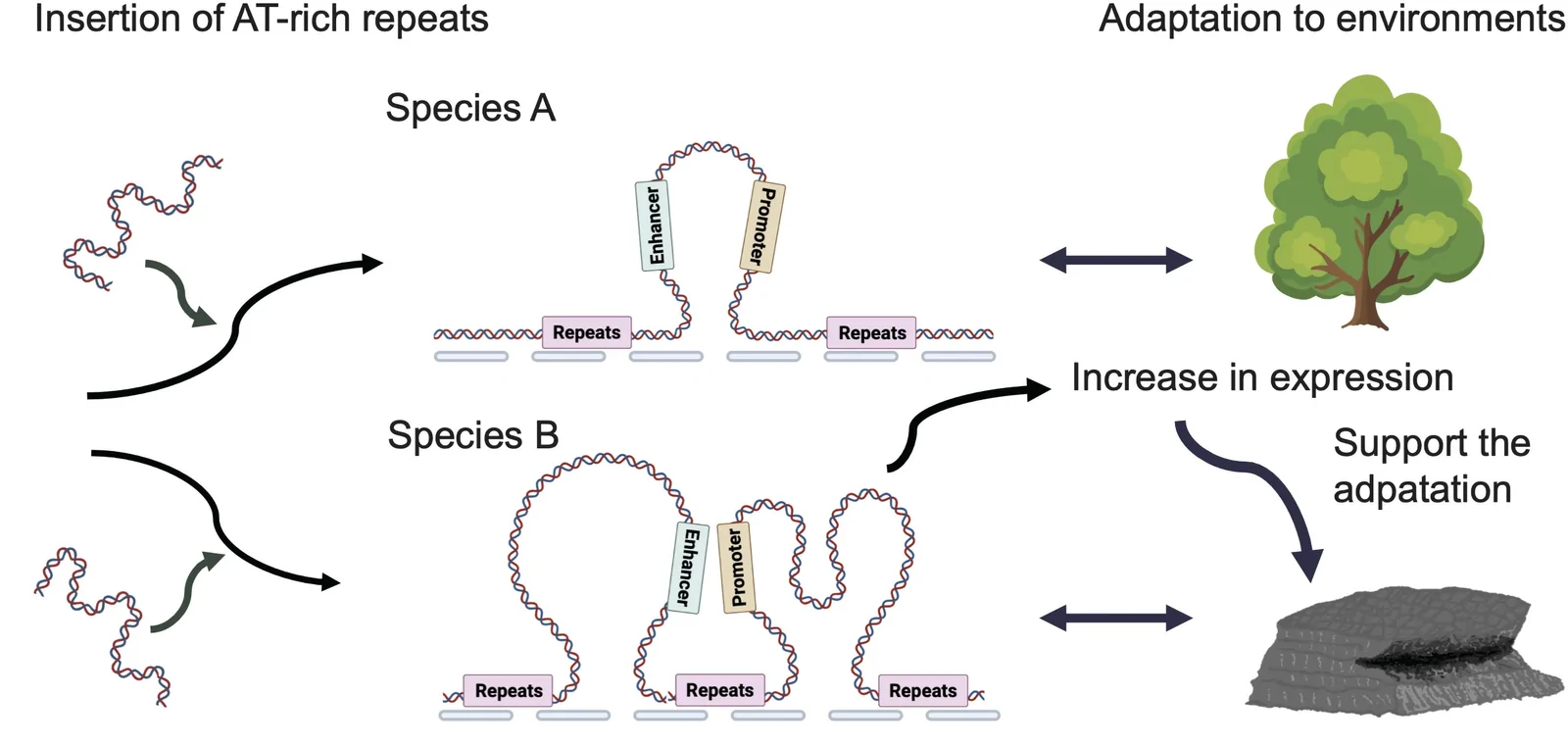
Hypothetical model illustrating how lineage-specific insertions of AT-rich sequences may influence chromatin organization and context-dependent regulation. Species A: candidate regulatory elements and target genes are positioned in a chromatin configuration that may limit their interaction. Species B: insertion or expansion of AT-rich isochores may alter local chromatin-loop organization, potentially changing interactions between candidate regulatory elements and nearby genes. This model is conceptual and remains to be tested by stress-condition expression profiling, chromatin-conformation assays, and functional analyses.

First, the spatial distribution and sequence composition of isochores resemble scaffold/matrix attachment regions (S/MARs), which organize chromatin into loops by anchoring DNA to the nuclear matrix (Torres et al. 2023). S/MAR-like domains have been described in *Neurospora crassa* (Reckard et al. 2024), and if *Annulohypoxylon* isochores serve a similar role, their lineage-specific placement could reshape local chromatin topology (Torres et al. 2023). Such architectural restructuring may modulate the responsiveness of nearby genes—particularly those involved in stress tolerance—without requiring changes to the genes themselves. This type of topological flexibility could offer a mechanism for lineage-specific regulatory variation (Fig. 6).

Second, AT-rich isochores may act as latent *cis*-regulatory elements. Repetitive sequences can carry promoter- or enhancer-like motifs capable of influencing nearby transcription (Sawaya et al. 2013; Muszewska et al. 2019). In *Annulohypoxylon*, isochores are enriched in predicted promoter motifs and exhibit low levels of DNA methylation, consistent with an accessible chromatin state. Using RNA-seq data generated under standard culture conditions, we found that genes flanking AT-rich isochores did not show a clear pattern of elevated basal expression relative to genome-wide expression levels. Thus, the current data do not support broad constitutive activation of neighboring genes under standard growth conditions. Instead, the motif enrichment and methylation patterns raise the possibility that these regions may influence gene regulation only under specific environmental or developmental contexts. This possibility remains to be tested directly with stress-condition expression profiling and chromatin-conformation analyses.

The physiological profile of the coastal strain *A. annulatoides* RYS0019—marked by distinct recovery after transient heat exposure and relatively strong performance under moderate salinity—leads us to propose that repeat-rich genome architecture may contribute to environmental flexibility (Fig. 6). In this model, AT-rich isochores may influence chromatin organization and create opportunities for context-dependent regulation without requiring major changes in gene content. This framework provides a testable link between genome architecture and phenotypic variation, but further work will be needed to evaluate it directly through stress-condition transcriptomics, chromatin-conformation assays, and functional perturbation of candidate AT-rich regions.

### Conclusion

This study suggests that physiological variation and repeat-associated genome architecture may both contribute to environmental flexibility in *A. annulatoides*. The coastal isolate RYS0019 showed a distinct stress-response profile relative to 5 bark-derived conspecific strains, while comparative genomics revealed widespread, lineage-specific AT-rich isochores across *Annulohypoxylon* genomes. These gene-poor, repeat-dense regions are largely embedded within conserved syntenic contexts and mostly uncoupled from large-scale contig rearrangements, supporting recurrent repeat-driven formation or expansion after species divergence. Their low methylation, enrichment for candidate promoter-associated motifs, and structural resemblance to S/MARs suggest potential roles in chromatin organization or context-dependent regulation. Although additional coastal isolates and stress-condition functional data will be needed to test this model directly, our findings highlight repeat-rich genome architecture as a dynamic feature of fungal genome evolution and a potential substrate for ecological flexibility beyond gene gain or loss.

## Supplementary Material

jkag192_Supplementary_Data

## Data Availability

All sequencing data generated in this study have been deposited in the NCBI Sequence Read Archive (SRA) under BioProject PRJNA1381326 (Supplementary Table 6). Sanger sequencing data for coastal fungal strains have been deposited in GenBank (Supplementary Table 1). The genome assembly of *A. annulatoides* is available from NCBI under accession JBWCIQ000000000.1. Individual SRA accession numbers are as follows: SRR36465265 (BioSample: SAMN54128988; *A. annulatoides*  YMJ2251, NGS WGS), SRR36465264 (BioSample: SAMN54128989; *A. annulatoides*  YMJ2252, NGS WGS), SRR36465263 (BioSample: SAMN54128990; *A. annulatoides*  RYS0019, ONT WGS), SRR36465262 (BioSample: SAMN54128991; *A. annulatoides*  RYS0019, NGS WGS), and SRR36465266 (BioSample: SAMN54128992; *A. annulatoides*  RYS0019, NGS RNA-seq). Supplemental material available at G3 online.
