# Supplementary material for: A marine-derived fungal genome of Annulohypoxylon annulatoides reveals AT-rich isochores with putative regulatory functions
Source: G3 (Bethesda). 2026 Jul 13;16(9):jkag192. doi: 10.1093/g3journal/jkag192 (PMC13535480; doi:10.1093/g3journal/jkag192)
Supplement: Supplementary file 1 [file jkag192_supplementary_data.zip › Supplementary_Information_G3-2026-406894.pdf]

Supplementary Information

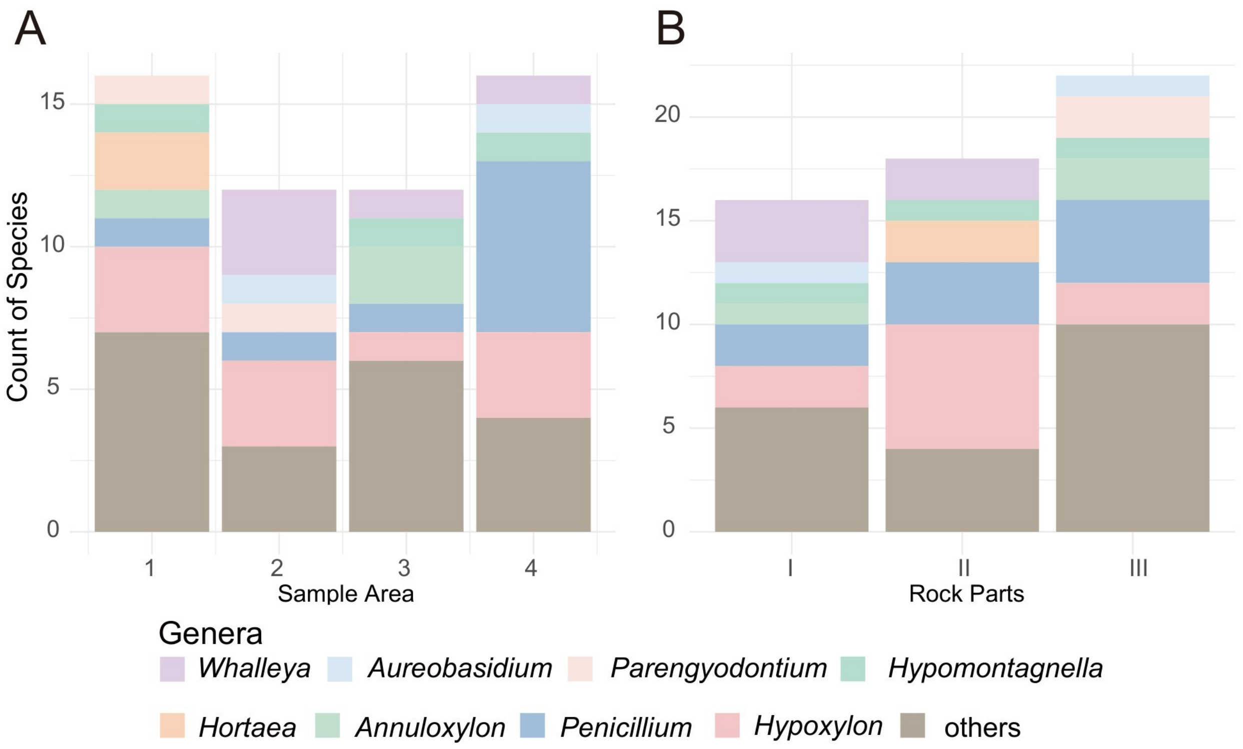

Supplementary Figure 1. Genus-level composition of culturable fungi across the Lailai rocky shore sampling design. (A) Stacked bar plots showing the number of isolates recovered per genus in each sampling area (Areas 1–4 along the transect from the vegetation line to the seaward edge). (B) Stacked bar plots showing the number of isolates recovered per genus across the three sampled rock microhabitats (I-III; corresponding to the upper surface, lateral side, and crevice, respectively). Each coloured segment denotes a genus, and bar height reflects total isolate counts per category.

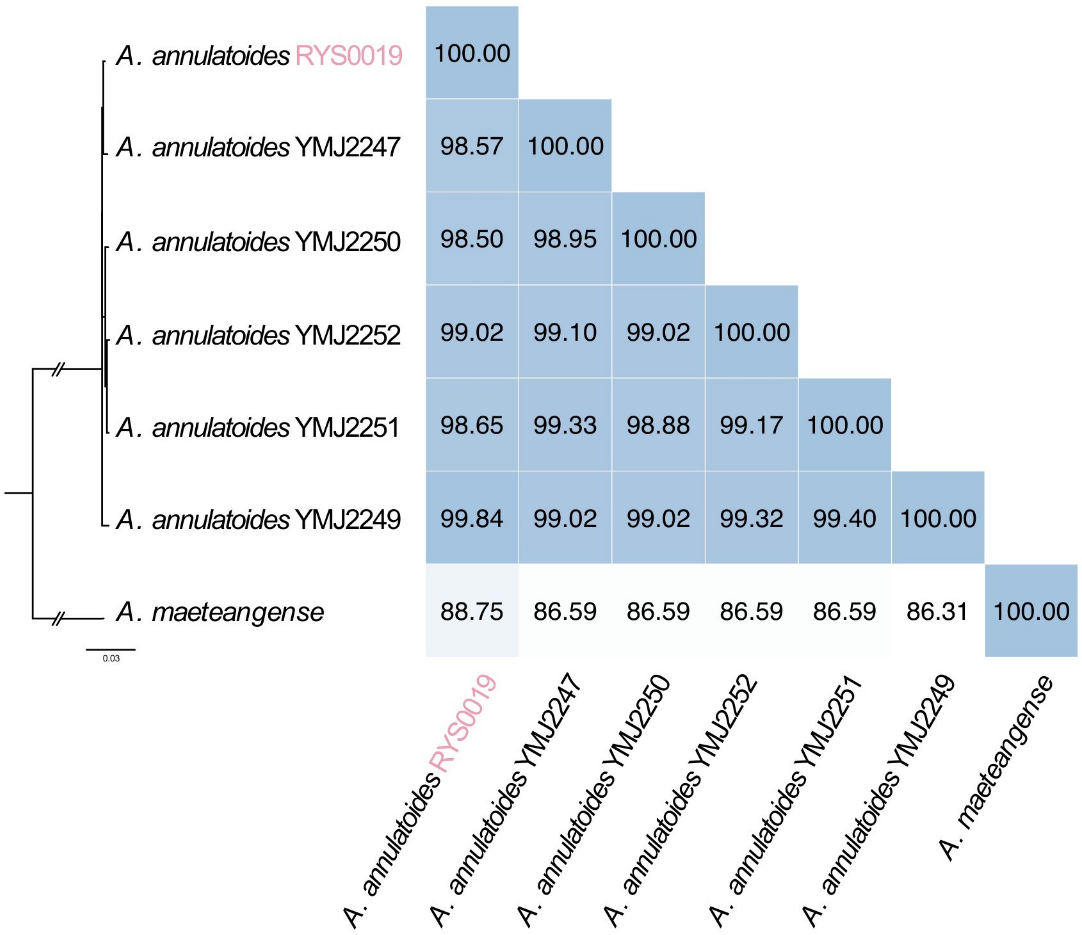

Supplementary Figure 2. Phylogenetic relationships and pairwise BLASTN sequence similarity among six *Annulohypoxylon annulatoides* strains of the ITS sequence.

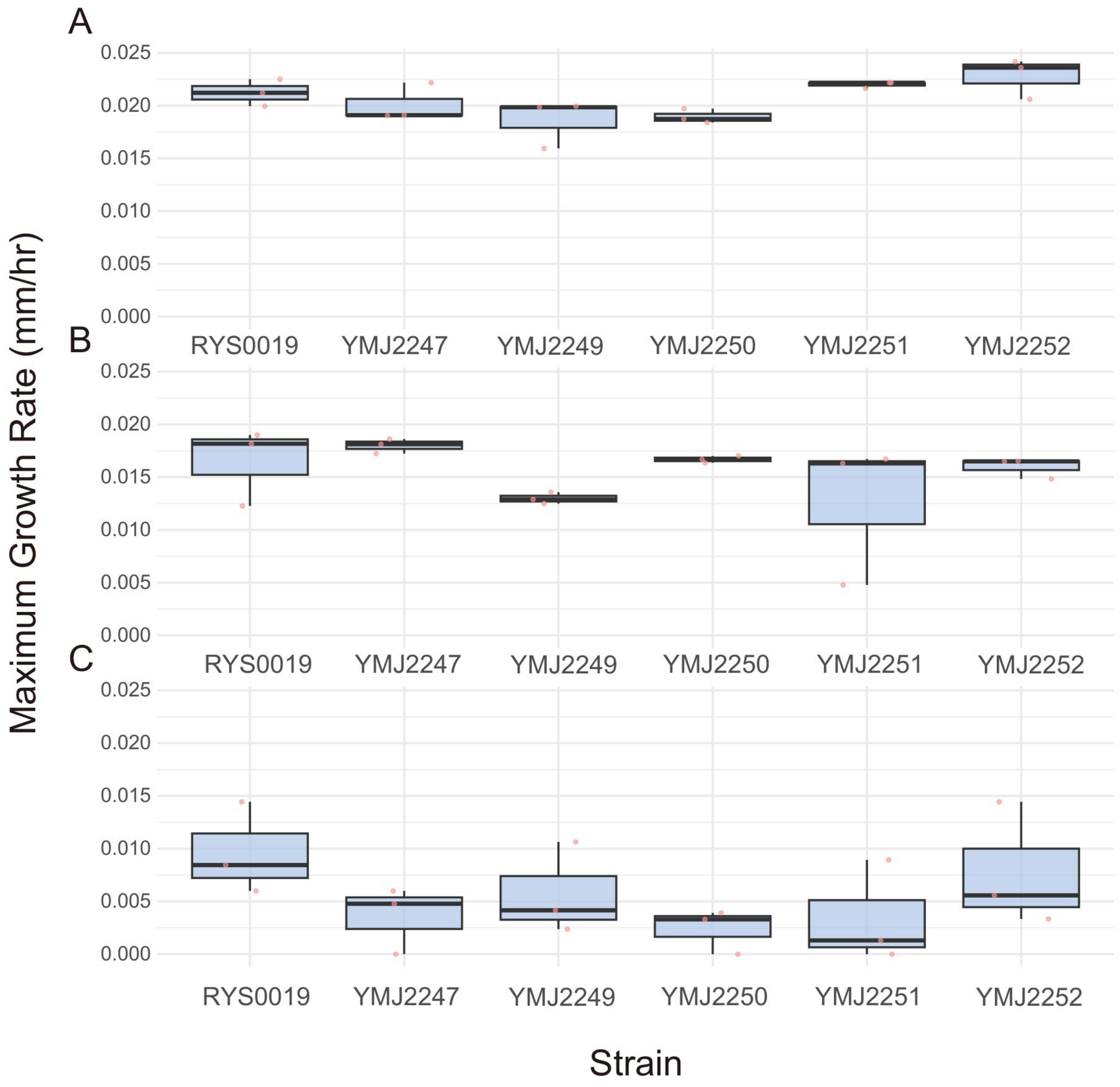

Supplementary Figure 3. Temperature-dependent growth performance of six *Annulohypoxylon annulatoides* strains. Boxplots show maximum growth rates of the coastal strain RYS0019 and five terrestrial strains (YMJ2247–YMJ2252) cultured in 0‰ NaCl medium at (A) 20 °C, (B) 30 °C, and (C) 40 °C. Points indicate individual biological replicates.

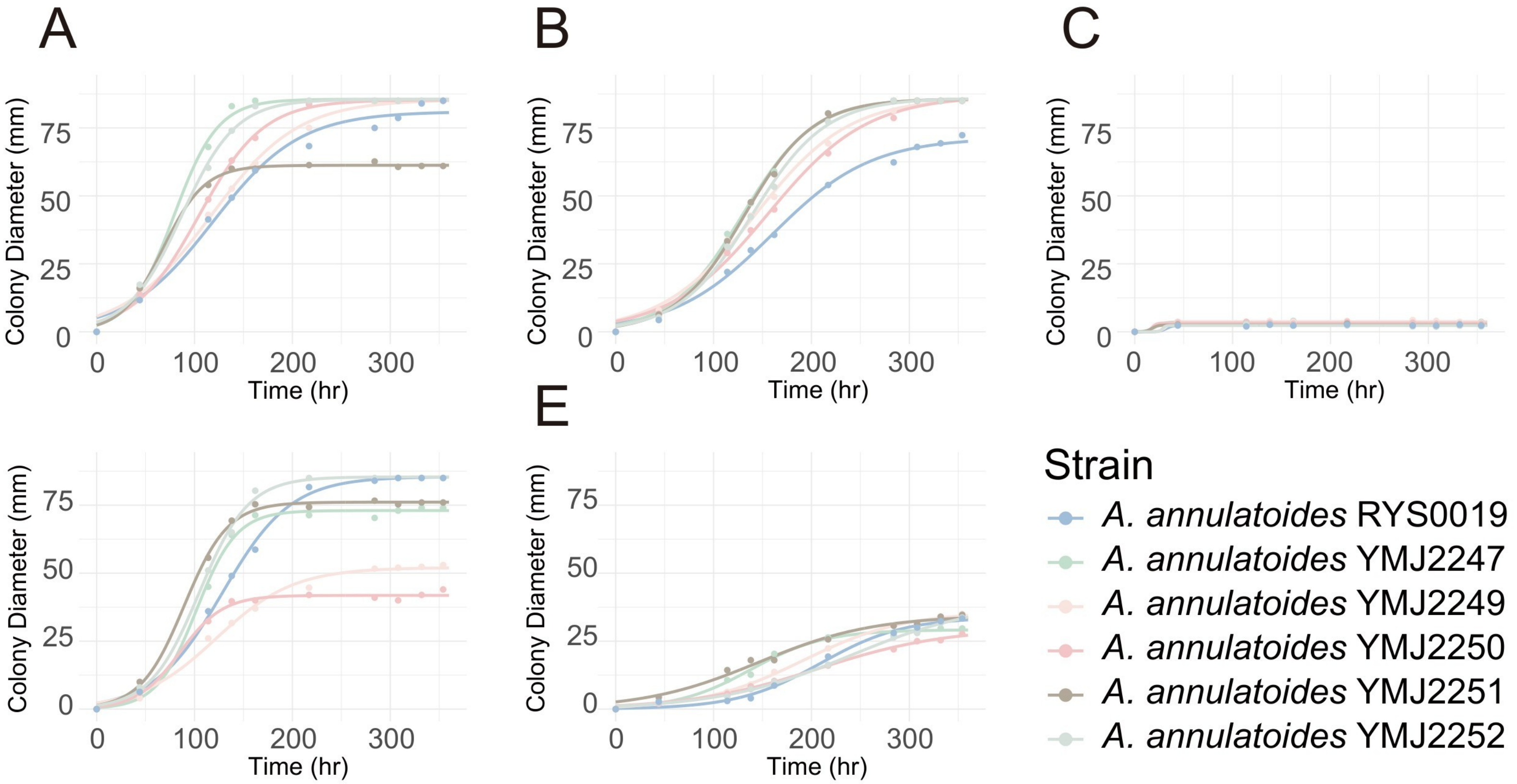

Supplementary Figure 4. Growth dynamics of six *Annulohypoxylon annulatoides* strains under varying temperature and salinity conditions. Time-course growth curves show hyphal expansion of the coastal strain RYS0019 and five terrestrial strains (YMJ2247–YMJ2252) cultured under five environmental conditions: (A) 30 °C, 0‰ NaCl; (B) 20 °C, 0‰ NaCl; (C) 40 °C, 0‰ NaCl; (D) 30 °C, 30‰ NaCl; and (E) 30 °C, 70‰ NaCl. Curves represent mean growth trajectories across biological replicates.

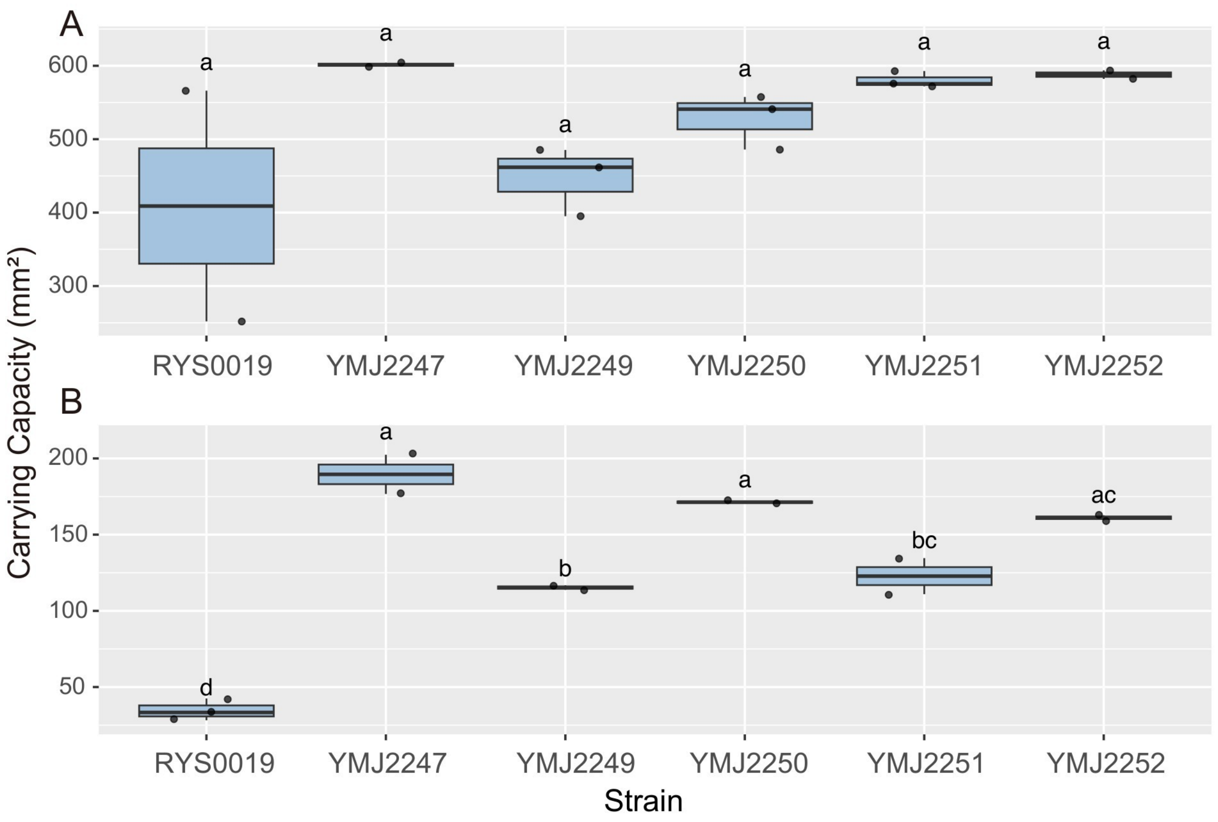

Supplementary Figure 5. Effects of UV stress on carrying capacity in six *Annulohypoxylon annulatoides* strains. Boxplots show carrying capacity (mm²) of the coastal strain RYS0019 and five terrestrial strains (YMJ2247–YMJ2252) under two conditions: (A) standard growth at 30 °C in 0‰ NaCl medium, and (B) following a one-day UV shock, with subsequent recovery at 30 °C in 30‰ NaCl medium. Letters above boxplots indicate statistically significant groupings based on one-way ANOVA followed by Tukey's post hoc test ($P < 0.05$).

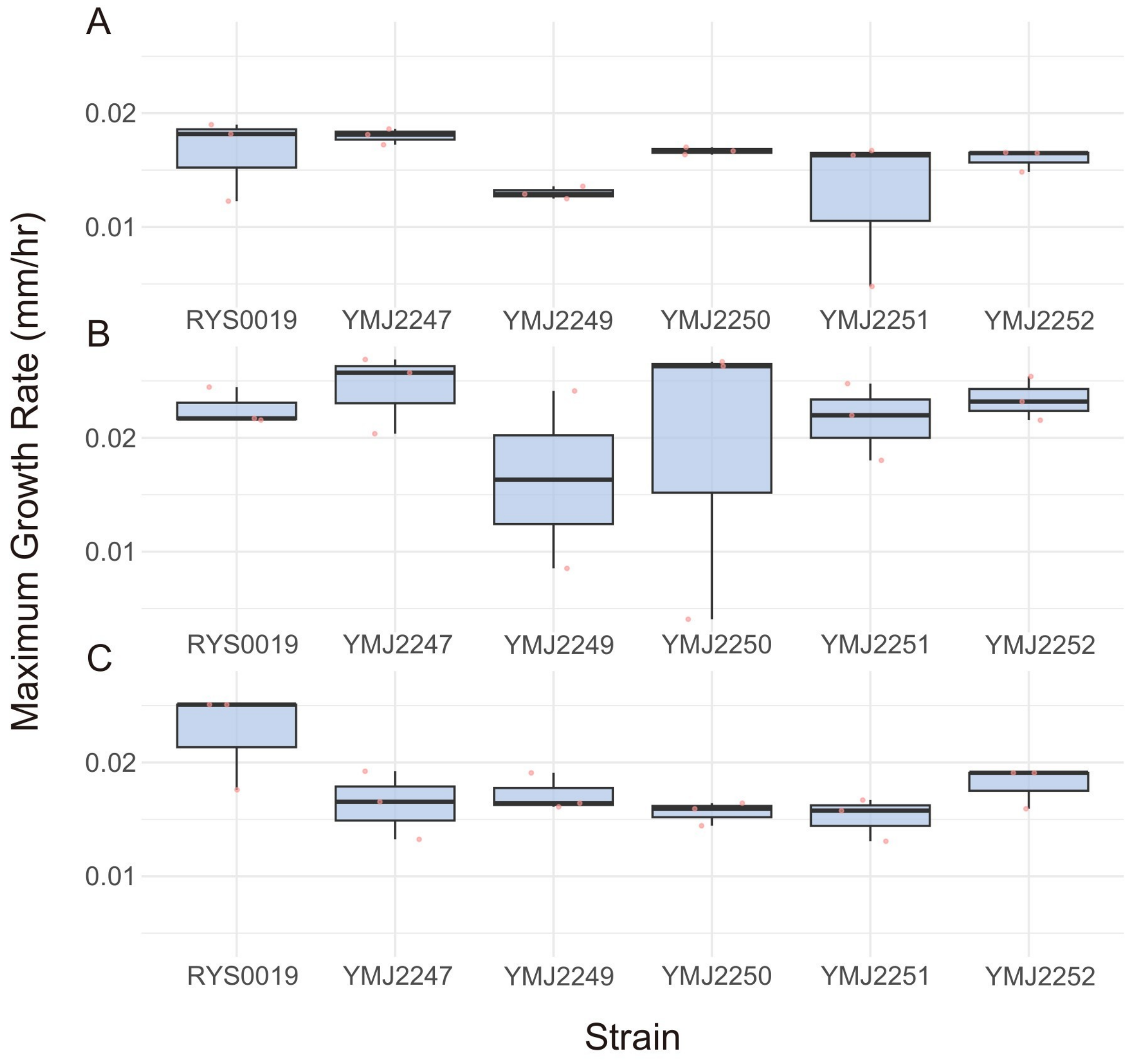

Supplementary Figure 6. Salinity-dependent growth performance of six *Annulohypoxylon annulatoides* strains at 30 °C. Boxplots show maximum growth rates (mm² hr⁻¹) of the coastal strain RYS0019 and five terrestrial strains (YMJ2247–YMJ2252) cultured at 30 °C under three salinity conditions: (A) 0‰ NaCl, (B) 30‰ NaCl, and (C) 70‰ NaCl. Points represent individual biological replicates.

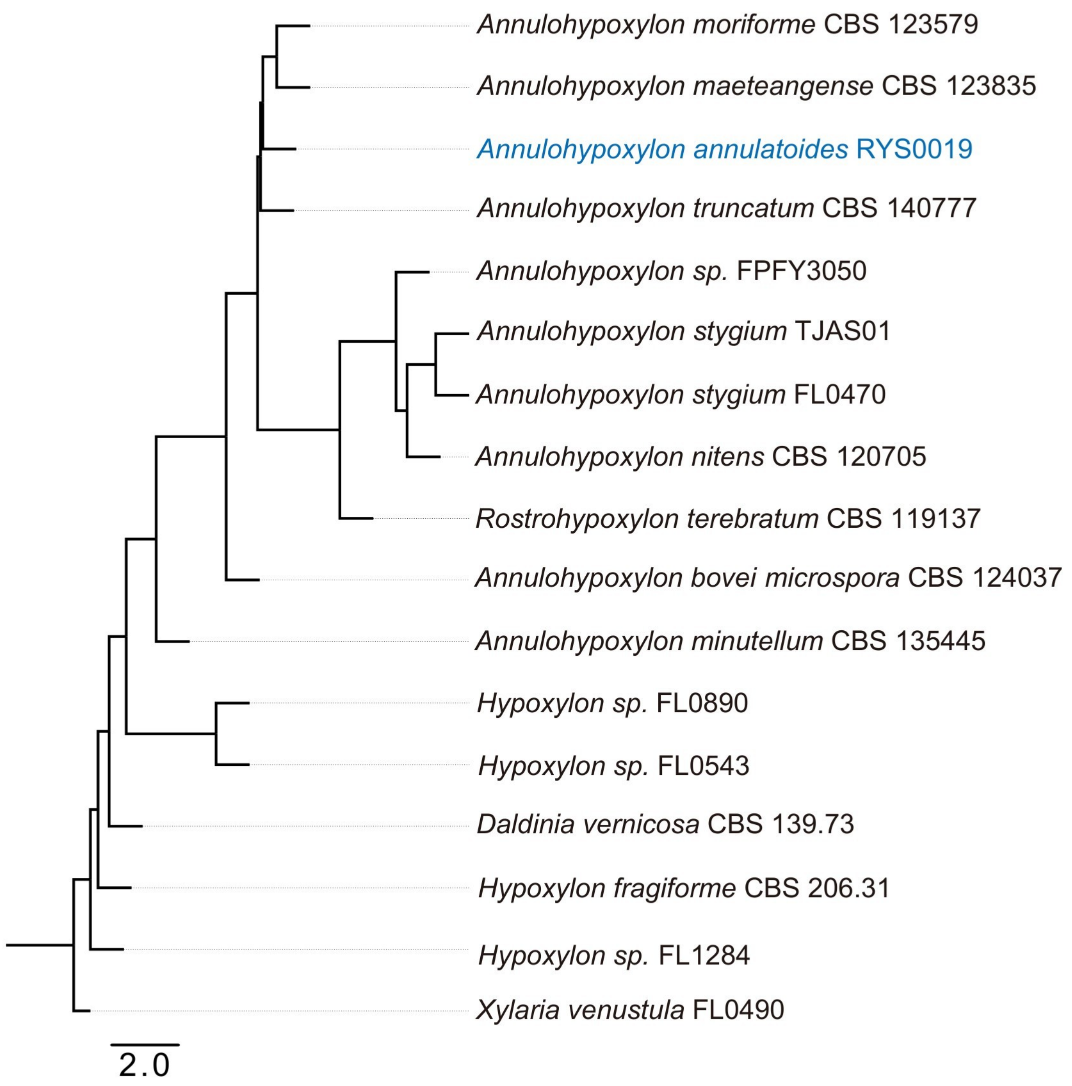

Supplementary Figure 7. Phylogenetic relationships among 16 Hypoxylaceae species, rooted with *Xylaria venustula* FL0490 as the outgroup. Node labels indicate ASTRAL local posterior probabilities. All branches have support values of 1.

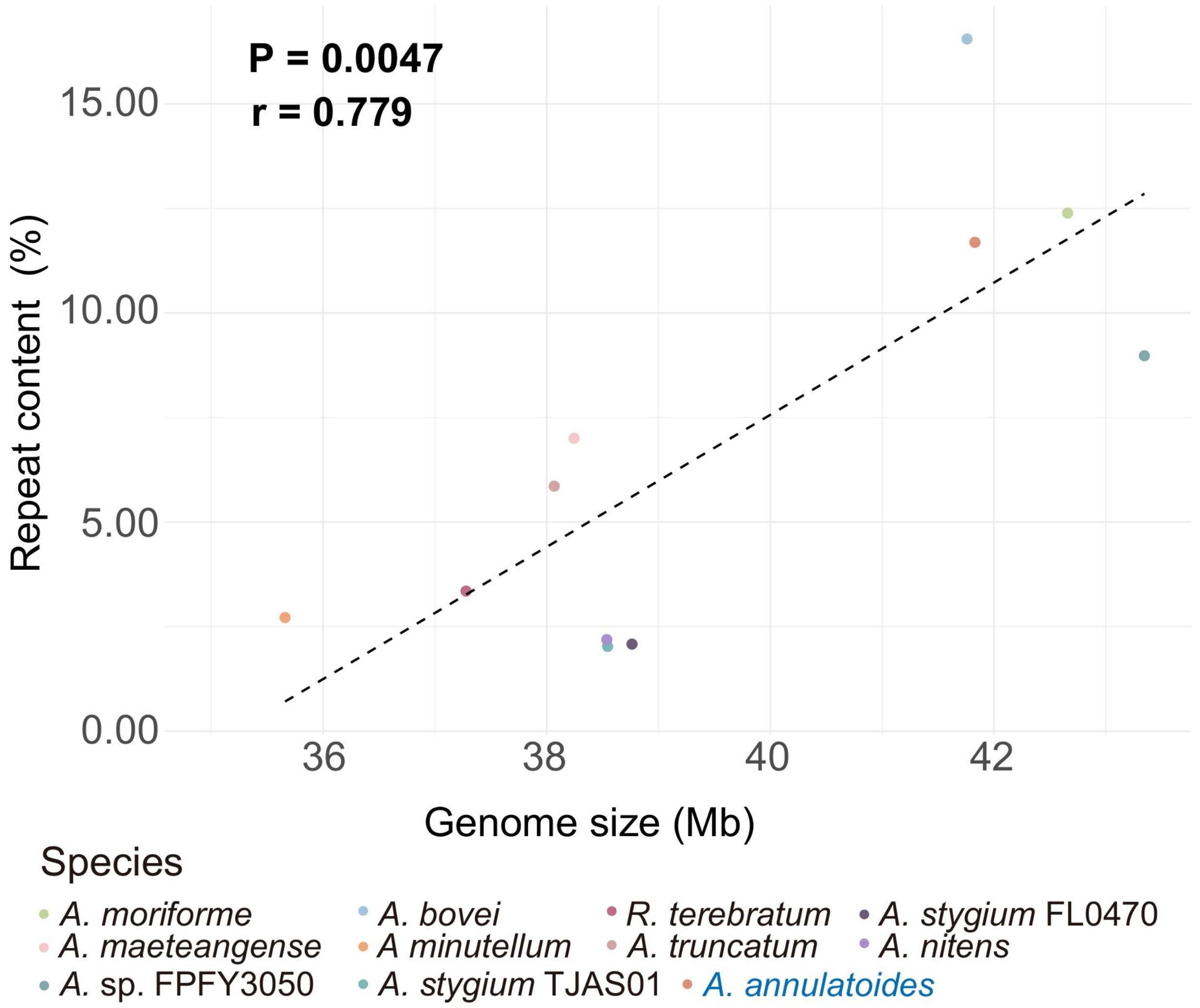

Supplementary Figure 8. Genome size scales with repeat content across *Annulohypoxylon* species. Scatterplot showing the relationship between total genome size (Mbp) and total repeat content (%) across 11 *Annulohypoxylon* species. Each point represents one species, coloured by taxon, with *A. annulatoides* highlightedfor reference.

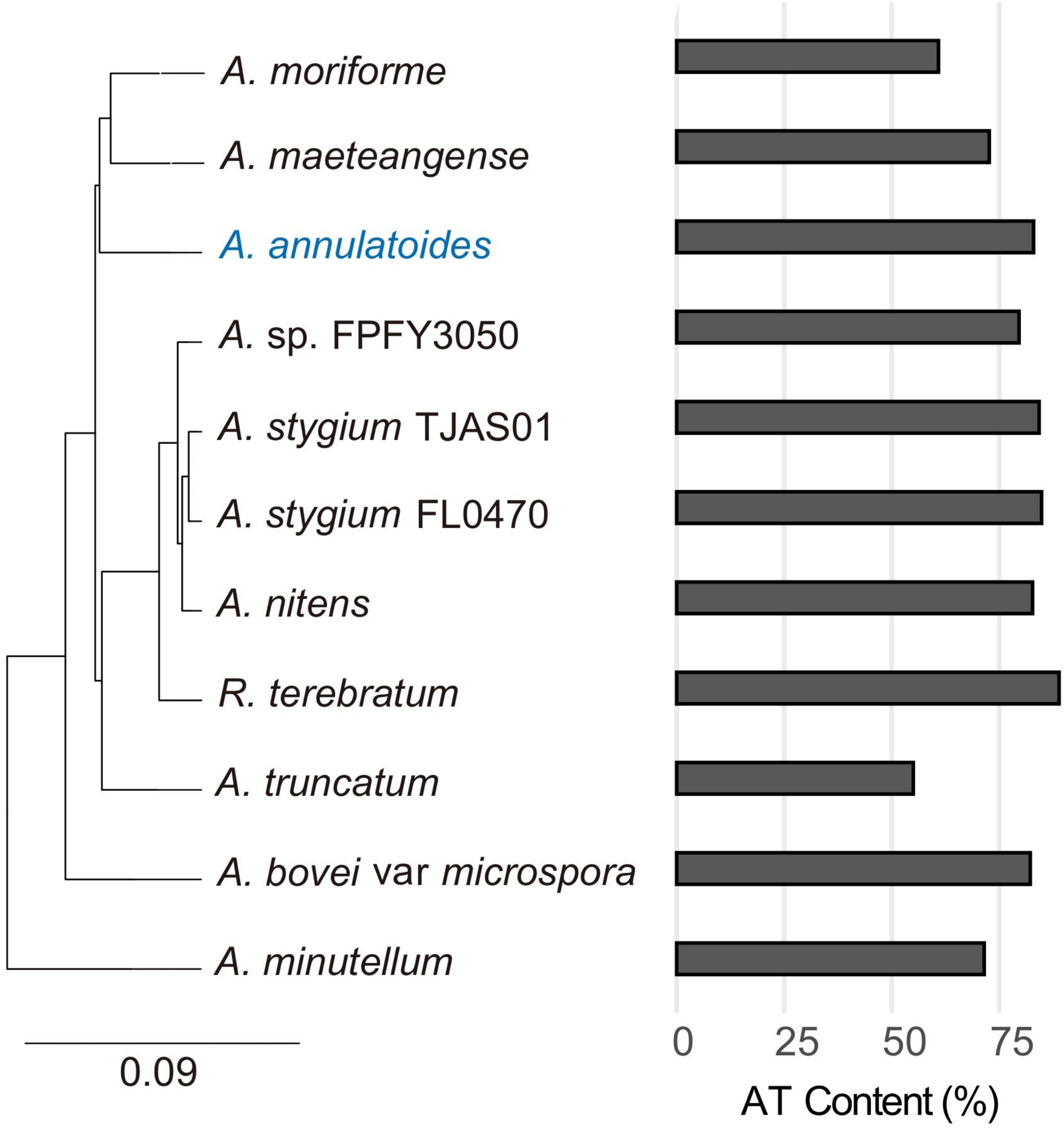

Supplementary Figure 9. AT content of repeat sequences across the genomes of 11 *Annulohypoxylon* species.

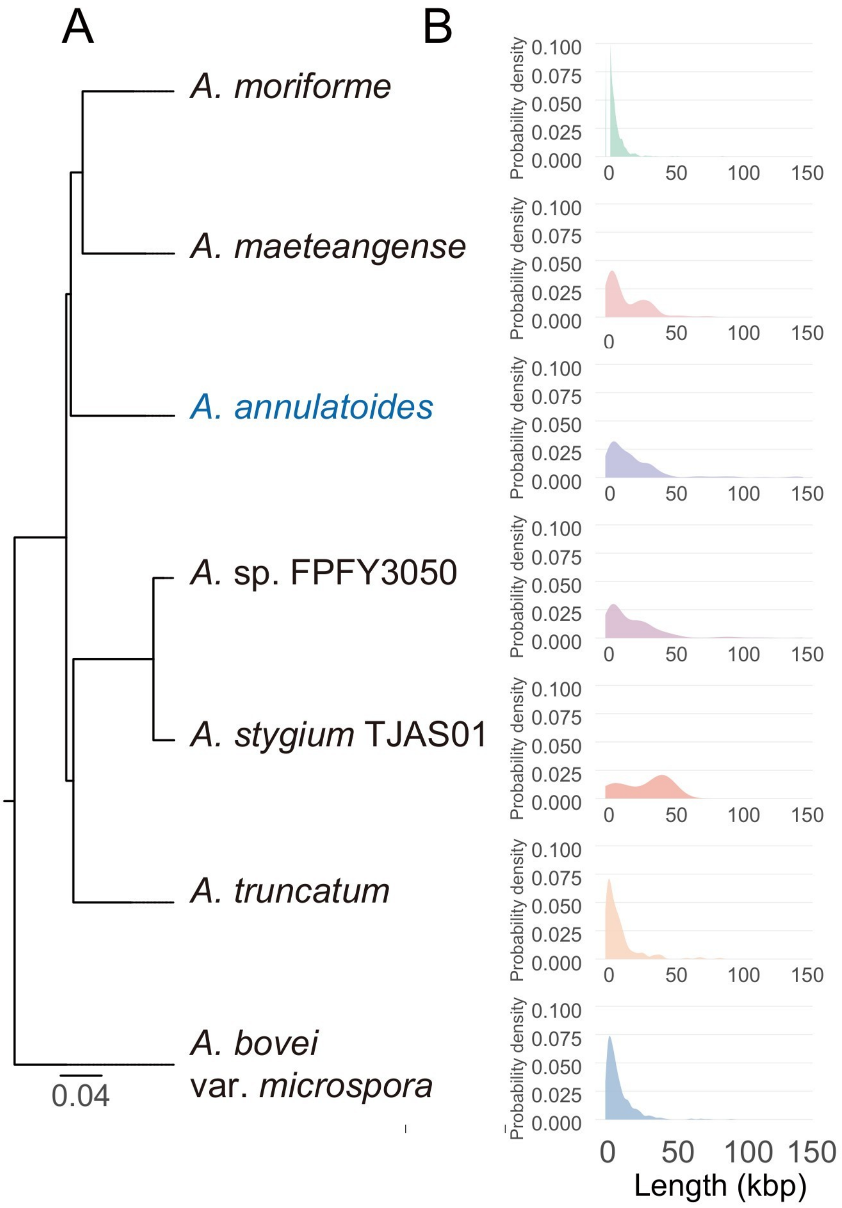

Supplementary Figure 10. Length distributions of AT-rich isochores across *Annulohypoxylon* species. (A) Phylogenetic relationships among the seven Hypoxylaceae species analysed, highlighting the placement of *A. annulatoides* relative to closely related taxa. (B) Kernel density estimates showing the length distributions of AT-rich isochores in *A. moriforme*, *A. maeteangense*, *A. annulatoides*, *A.* sp. FPYF3050, *A. stygium* TJAS01, *A. truncatum*, and *A. bovei* var. *microspora*.

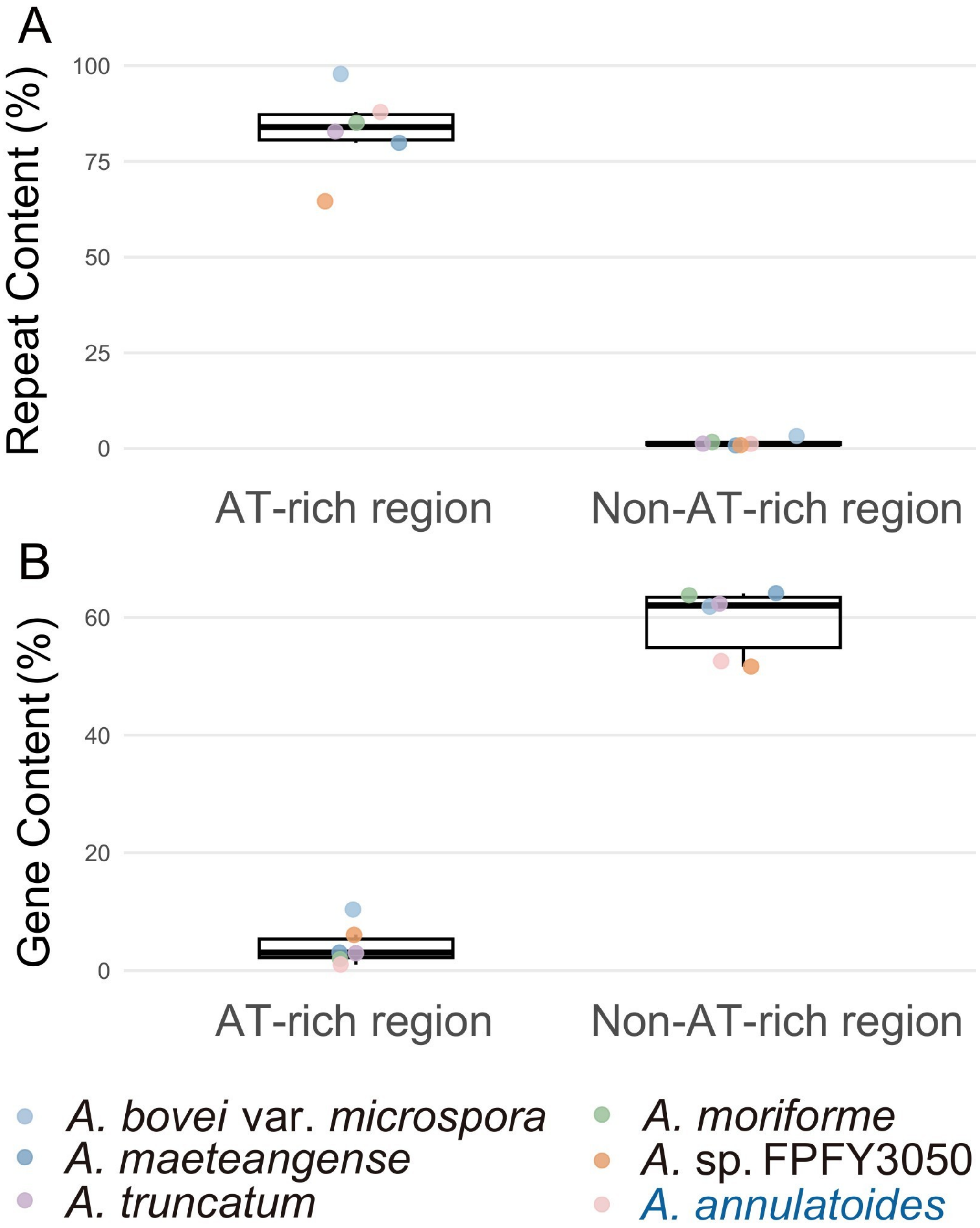

Supplementary Figure 11. Comparison of (A) repeat coverage (B) gene coverage between genomic regions with AT content > 60% and those with AT content < 60% across six *Annulohypoxylon* species.

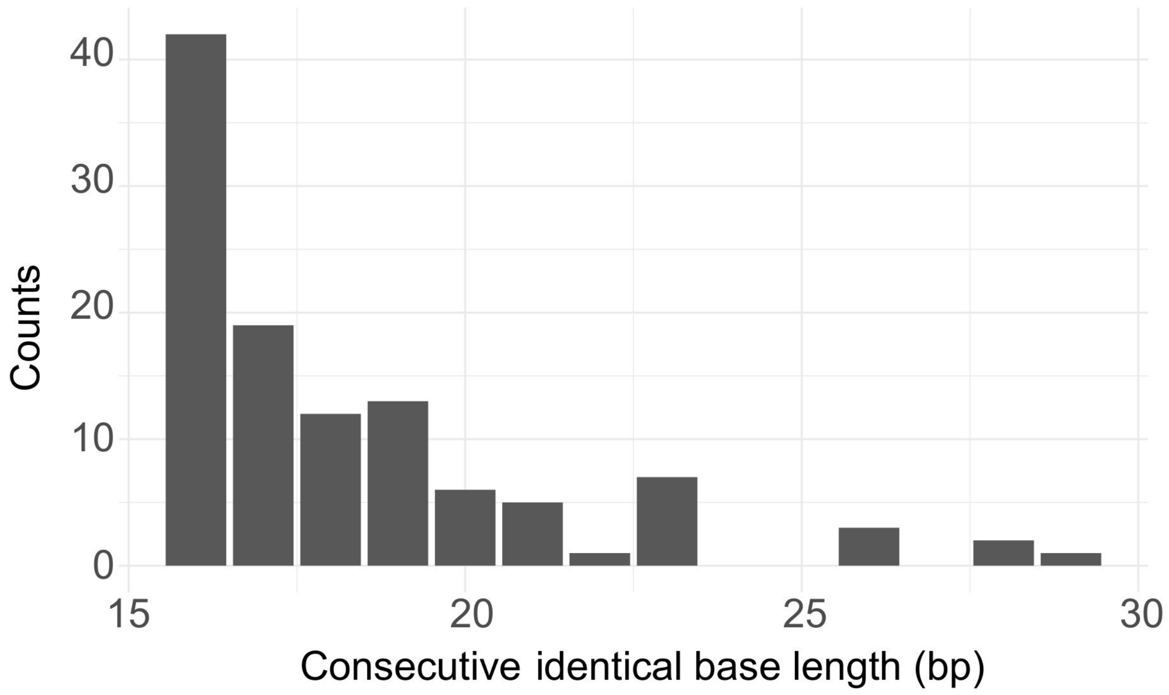

Supplementary Figure 12. Distribution of homopolymers longer than 15 bp in AT-rich isochores of *A. annulatoides* RYS0019.

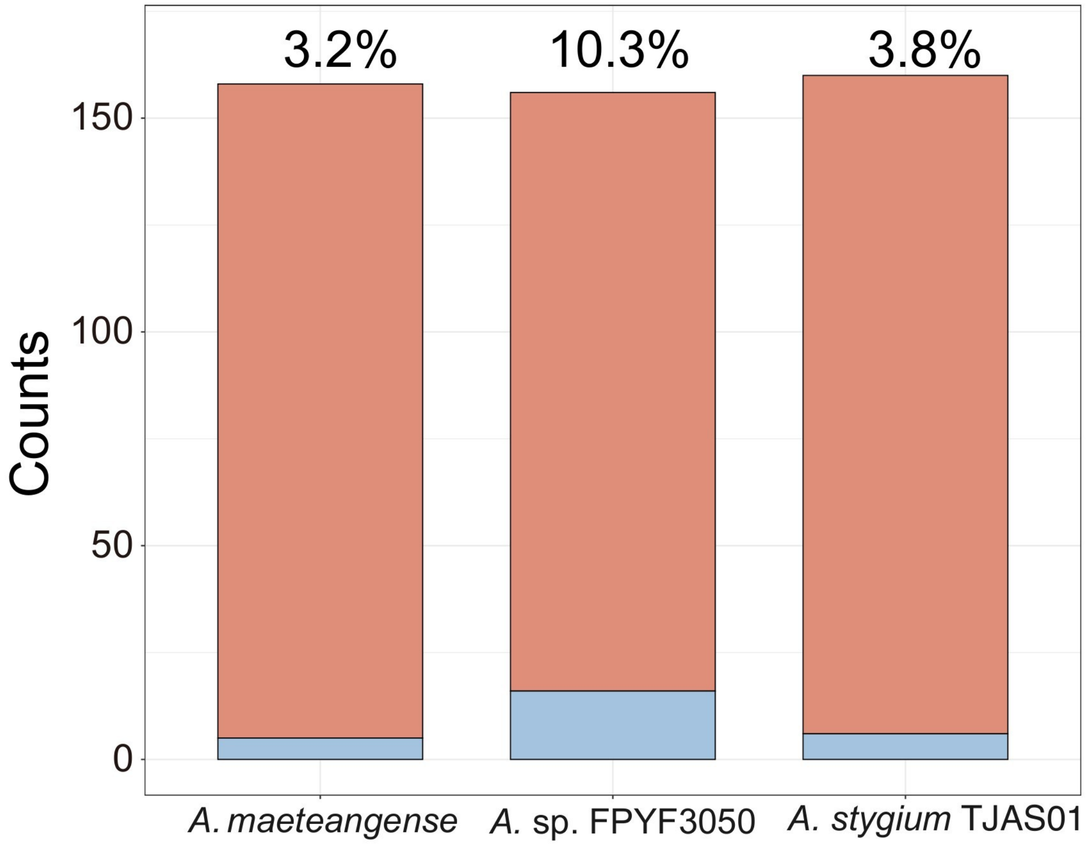

Supplementary Figure 13. Limited positional conservation of AT-rich isochores across *Annulohypoxylon* species. Stacked bar plots show intergenic regions in three *Annulohypoxylon* species (*A. maeteangense*, *A.* sp. FPYF3050, and *A. stygium* TJAS01) that either share an AT-rich isochore with *A. annulatoides* (blue) or lack an isochore at the corresponding position (salmon). Percentages above bars indicate the proportion of intergenic regions with shared isochores.

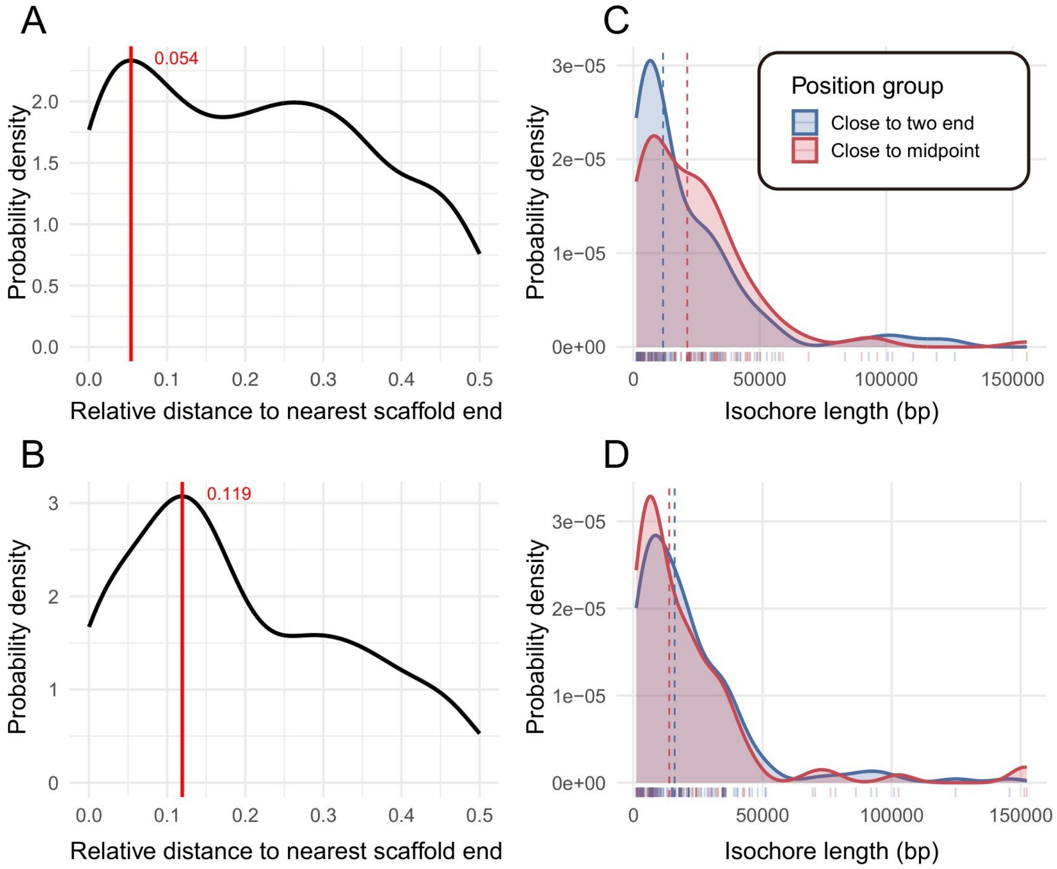

Supplementary Figure 14. Length distributions of AT-rich isochores along chromosomes. (A, B) Kernel density distributions showing the relative positions of AT-rich isochores in *A.* sp. FPYF3050 (A) and *A. annulatoides* (B), measured as the normalized distance to the nearest scaffold end (0 = scaffold end; 0.5 = scaffold midpoint). Vertical dashed lines indicate median positions, (C, D) Kernel density distributions of AT-rich isochore lengths in *A.* sp. FPYF3050 (C) and *A. annulatoides* (D), stratified by positional category. Isochores with relative distance ≤ 0.25 were classified as near-terminal, whereas those with distance > 0.25 were classified as central. Dashed lines mark median isochore lengths for each group.

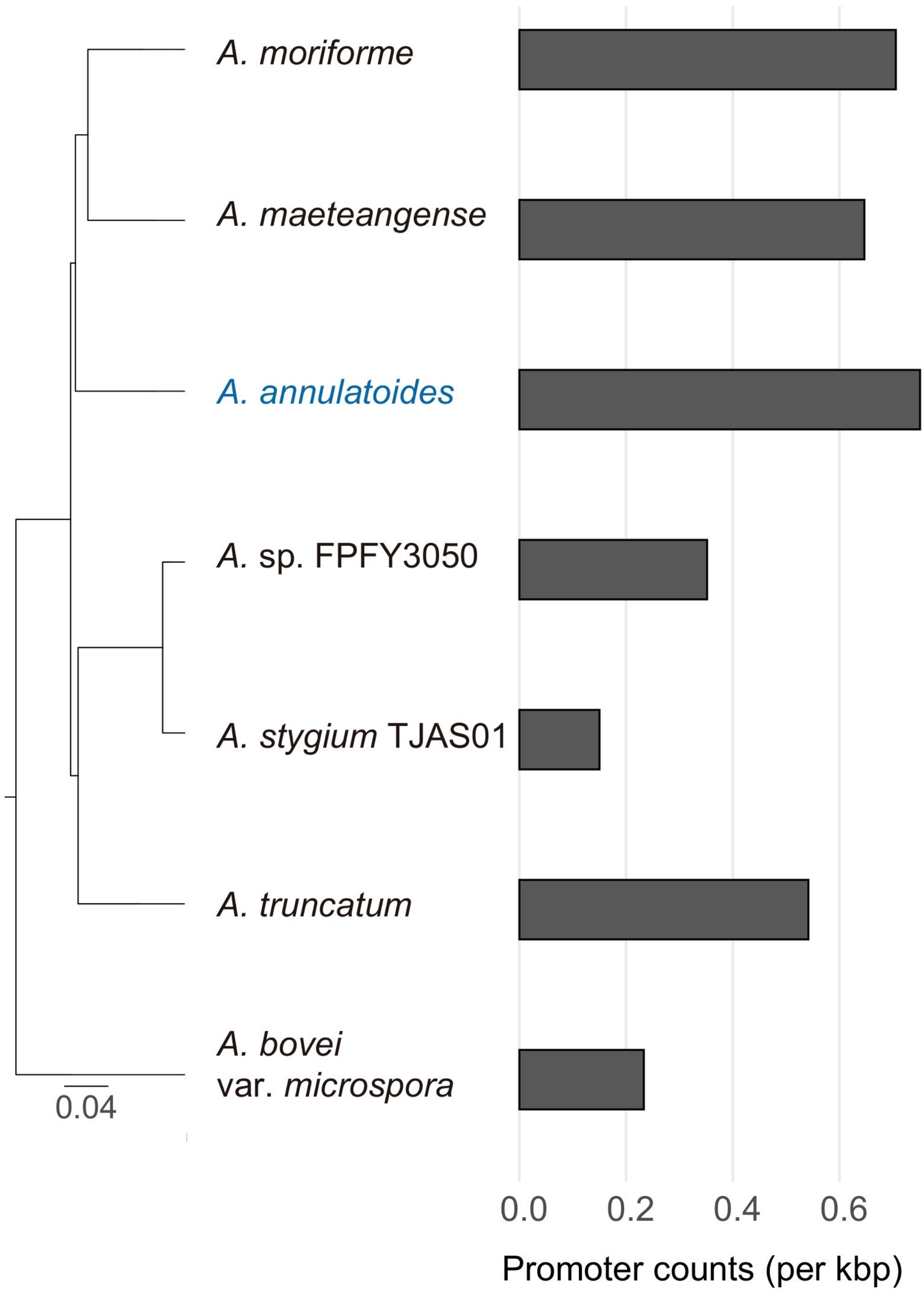

Supplementary Figure 15. Promoter-associated motif density in AT-rich and random genomic regions of three *Annulohypoxylon* species. Promoter-associated motifs were identified using FIMO based on JASPAR fungi transcription factor binding profiles. Bars show motif density per kb in AT-rich regions and 1,000 size-matched randomly sampled genomic regions. p values were calculated by permutation tests.

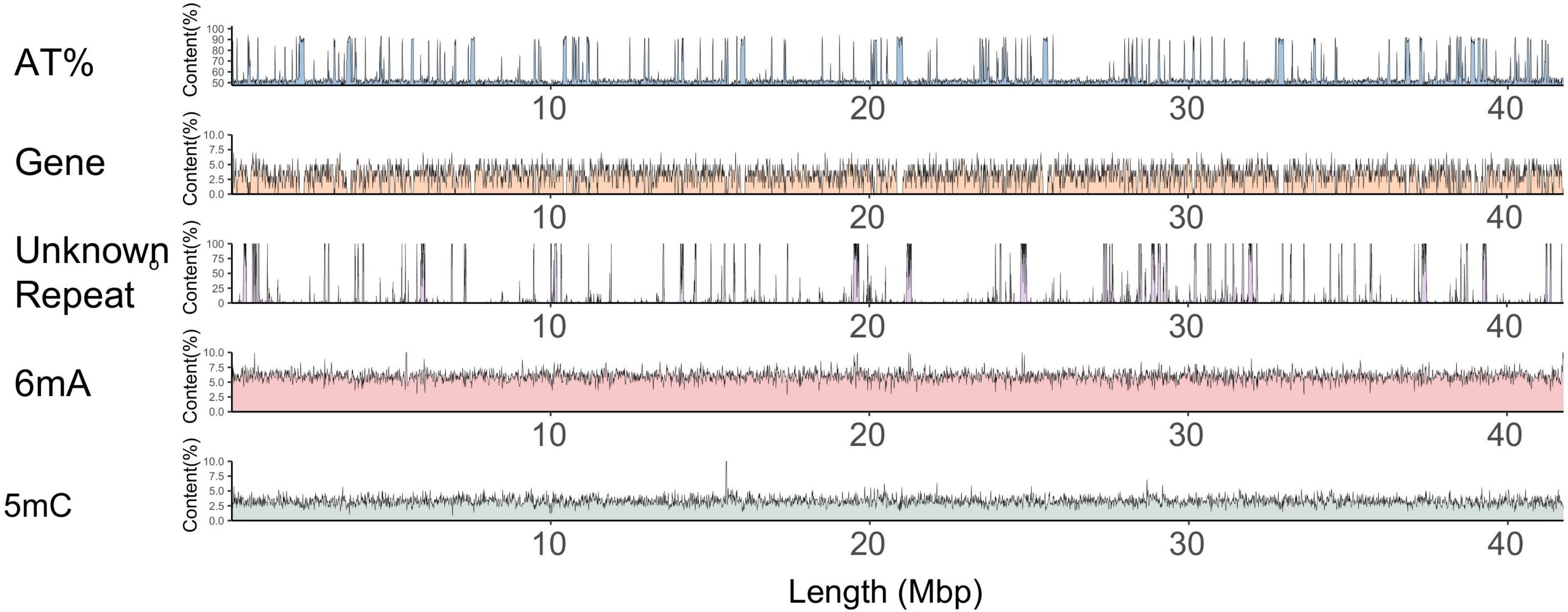

Supplementary Figure 16. Genome-wide distribution of sequence composition and epigenetic features in *Annulohypoxylon annulatoides*. Tracks show genome-wide profiles of gene density, repeat density, AT content, N6-methyldeoxyadenosine (6mA) methylation, and 5-methylcytosine (5mC) methylation across the *A. annulatoides* genome, calculated using a 10-kb sliding window.

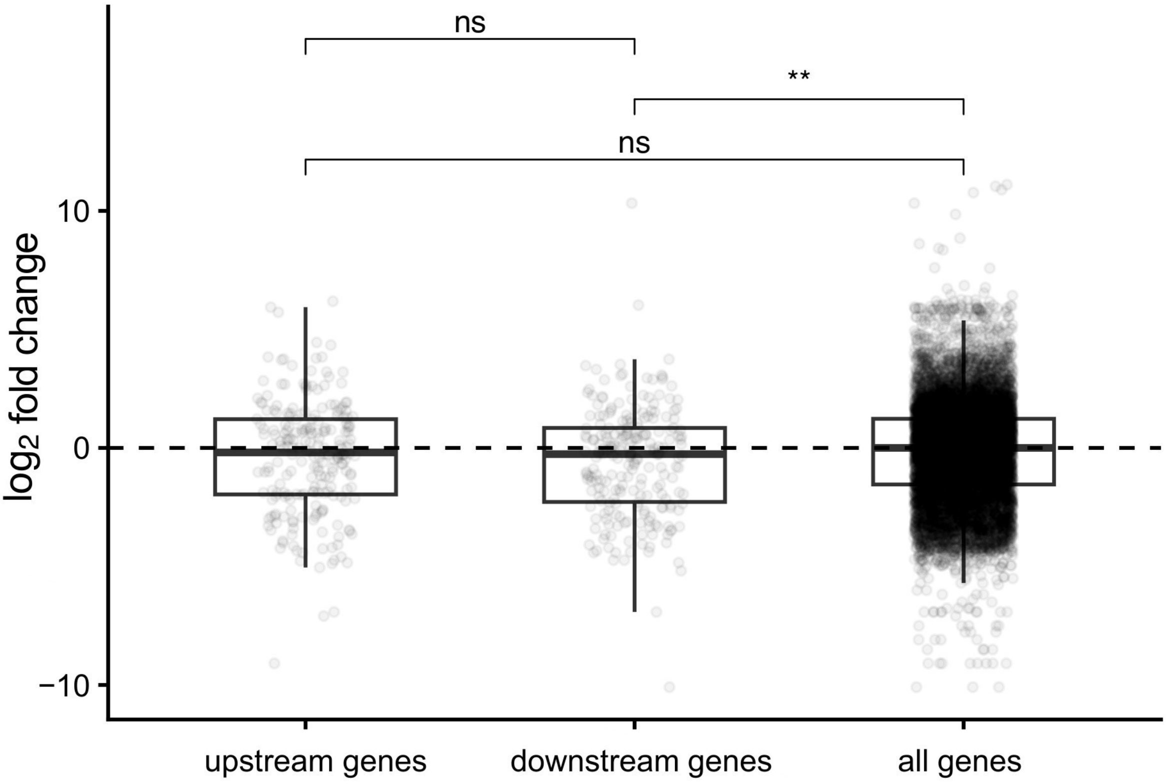

Supplementary Figure 17. Basal expression of genes flanking AT-rich isochores under standard growth conditions.

RNA-seq reads generated from Annulohypoxylon annulatoides RYS0019 under standard growth conditions were mapped to the reference genome using STAR. Gene-level expression values were used to compare all genes with genes immediately upstream or downstream of AT-rich isochores. Expression values are shown as $\log_2$-transformed expression relative to the genome-wide median expression level. No stress-treatment comparison was performed. Statistical significance was assessed using Student's t-test (ns, not significant; **$P < 0.01$; ***$P < 0.001$).

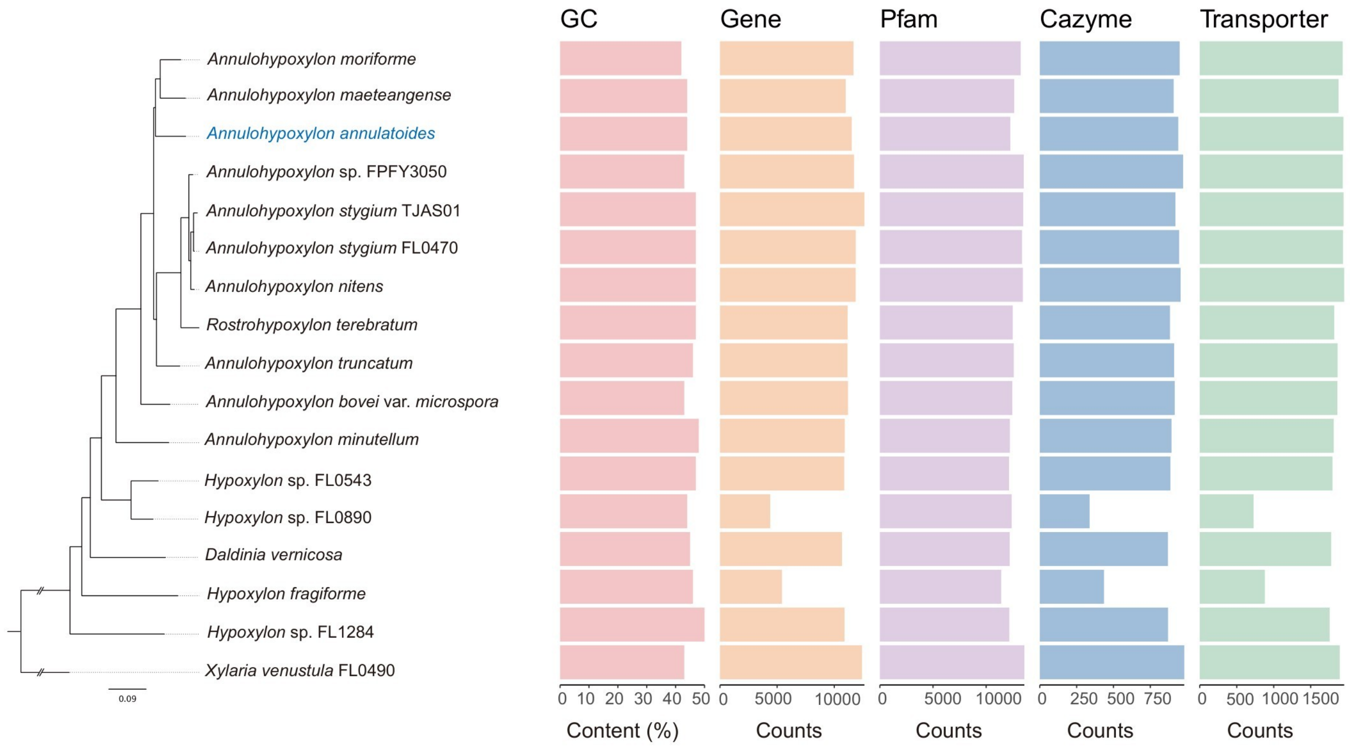

Supplementary Figure 18. Conserved gene and functional repertoire across Hypoxylaceae genomes. (A) Phylogenetic relationships among 16 Hypoxylaceae species with *Xylaria venustula* FL0490 shown as the outgroup; *Annulohypoxylon annulatoides* is highlighted for reference. (B) Comparative profiles of genome composition and functional gene content across species, including GC content (%), total gene counts, Pfam domain counts, carbohydrate-active enzymes (CAZymes), and predicted transporters.

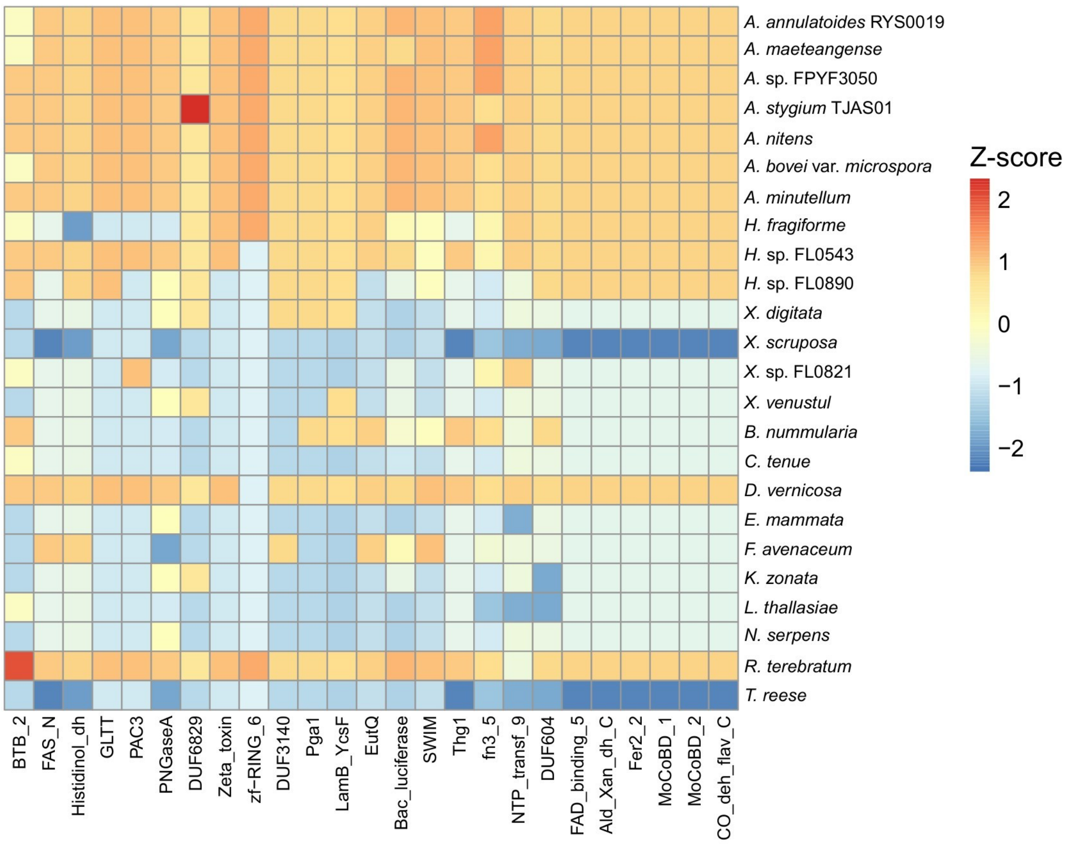

Supplementary Figure 19. Protein families enriched in Hypoxylaceae relative to other ascomycete fungi. Heatmap showing protein families that are significantly more abundant in Hypoxylaceae species compared with non-Hypoxylaceae ascomycete outgroups. Protein families were identified using a Wilcoxon rank-sum test with Benjamini–Hochberg correction (adjusted $P < 0.05$).
